# Integrated analysis of microRNAs, circular RNAs, long non-coding RNAs, and mRNAs revealed competing endogenous RNA networks involved in brown adipose tissue whitening in rabbits

**DOI:** 10.1186/s12864-022-09025-2

**Published:** 2022-11-28

**Authors:** Kun Du, Xue Bai, Li Chen, Yu Shi, Hao-ding Wang, Ming-cheng Cai, Wen-qiang Sun, Jie Wang, Shi-yi Chen, Xian-bo Jia, Song-jia Lai

**Affiliations:** 1 Farm Animal Genetic Resources Exploration and Innovation Key Laboratory of Sichuan Province, Sichuan Agricultural University, 211# Huimin Road, Wenjiang, 611130 Sichuan China; 2 College of Landscape Architecture and Life Science/Institute of Special Plants, Chongqing University of Arts and Sciences, Chongqing, China

**Keywords:** BAT, Rabbits, Whitening, miRNA, circRNA, ceRNA

## Abstract

**Background:**

The brown adipose tissue (BAT) is a target for treating obesity. BAT losses thermogenic capacity and gains a “white adipose tissue-like” phenotype (“BAT whitening”) under thermoneutral environments, which is a potential factor causing a low curative effect in BAT-related obesity treatments. Circular RNAs (circRNAs) and long non-coding RNAs (lncRNAs) can act as competing endogenous RNAs (ceRNA) to mRNAs and function in various processes by sponging shared microRNAs (miRNAs). However, the roles of circRNA- and lncRNA-related ceRNA networks in regulating BAT whitening remain litter known.

**Results:**

In this study, BATs were collected from rabbits at day0 (D0), D15, D85, and 2 years (Y2). MiRNA-seq was performed to investigate miRNA changes during BAT whitening. Then, a combined analysis of circRNA-seq and whole-transcriptome sequencing was used for circRNA assembly and quantification during BAT whitening. Our data showed that 1187 miRNAs and 6204 circRNAs were expressed in the samples, and many of which were identified as significantly changed during BAT whitening. Target prediction showed that D0-selective miRNAs were significantly enriched in the Ras, MAPK, and PI3K-Akt signaling pathways, and Y2-selective miRNAs were predicted to be involved in cell proliferation. The cyclization of several circRNAs could form novel response elements of key thermogenesis miRNAs at the back-splicing junction (BSJ) sites, and in combination with a dual-luciferase reporter assay confirmed the binding between the BSJ site of novel_circ_0013792 and ocu-miR-378-5p. CircRNAs and lncRNAs have high cooperativity in sponging miRNAs during BAT whitening. Both circRNA-miRNA-mRNA and lncRNA-miRNA-mRNA triple networks were significantly involved in immune response-associated biological processes. The D15-selective circRNA might promote BAT whitening by increasing the expression of *IDH2.* The Y2-selective circRNA-related ceRNA network and lncRNA-related ceRNA network might regulate the formation of the WAT-like phenotype of BAT via MAPK and Ras signaling pathways, respectively.

**Conclusions:**

Our work systematically revealed ceRNA networks during BAT whitening in rabbits and might provide new insight into BAT-based obesity treatments.

**Supplementary Information:**

The online version contains supplementary material available at 10.1186/s12864-022-09025-2.

## Background

Obesity is a growing public health problem worldwide, which is caused by over-accumulation of fat in white adipose tissue (WAT) [1]. WAT is composed of white adipocytes that contain unilocular and big lipid droplets and mainly functions in energy storage, hormone secretion, and immune response [2, 3]. Except for WAT, there is another type of adipose tissue, brown adipose tissue (BAT). BAT plays a key role in thermogenesis through uncoupling respiration controlled by uncoupling protein 1 (UCP1) [4]. BATs are composed of brown adipocytes containing multilocular lipid droplets and can be further classified into two types, including the classical BATs that were mainly distributed in the interscapular regions of many newborn mammals and the induced BATs that were generated from WAT depots after being stimulated by prolonged cold exposure (the latter were also called beige fat) [5]. Because BAT activity positively correlates with the resting metabolic rate and negatively correlates with the BMI and fat content of the total body, it is a target for treating obesity [6–8].

Although the virtues of BAT in body metabolisms, the classical BAT loses thermogenic phenotype and covert to “WAT-like” adipose tissues under thermoneutral conditions, which was also known as “BAT whitening” [9, 10]. BAT whitening was considered a factor that promotes the development of obesity and other metabolic disorders and is a potential factor causing low curative effect in BAT-related obesity treatments [11, 12]. Interrogating the mechanisms of BAT whitening is the base for utilizing BAT to treat obesity. Previous studies have revealed that the prolonged adaptation to thermoneutrality accounts for the BAT whitening and *de novo* lipogenesis involved in the process [12]. On the transcription level, the exciting progress has identified several transcription factors (TFs), such as Nrf1 [13], ChREBP [12], and IFNG [14], could promote BAT whitening. While PPARA (a BAT-selective TF) could counter BAT whitening [15]. On the post-transcription level, our previous study has identified a set of long non-coding RNAs (lncRNAs) that were involved in the BAT whitening using whole-transcriptome sequencing [16]. Nevertheless, our knowledge of post-transcriptional regulation of BAT whitening remains at its infancy.

The interaction of different types of RNA is ubiquitous during the post-transcriptional process [17]. Circular RNAs (circRNA) are a novel class of covalently closed and single-stranded endogenous non-coding RNAs (ncRNAs), which play important roles in the various processes, such as tissue development [18, 19], response to external stimulus [20], and cancer formation [21]. CircRNAs can act as competing endogenous RNAs (ceRNAs), or miRNA sponges, to communicate with each other by competing for miRNA-binding through common miRNA response elements (MREs) [22]. Several circRNAs have been identified as ceRNAs in the regulation of fat biology, such as the ciRS-133 in promoting beige fat development [23], the CircSAMD4A in regulating preadipocyte differentiation [24], and the circArhgap5-2 in maintaining lipid biosynthesis and metabolism [25]. Similar to circRNA, recent studies have revealed that lncRNAs could also act as ceRNAs to mRNAs by sponging miRNAs [26]. Thus, the circRNAs, lncRNAs, miRNAs, and mRNAs could form complex miRNA-centered regulatory ceRNA networks in post-transcriptional regulation. Understanding the ceRNA networks poses a great opportunity to clarify the post-transcriptional regulation of BAT whitening.

Mice are a popular animal model for investigating BAT, of which the classical BAT persists into adult life [27, 28]. The classical BATs of rabbits almost disappear in adults, which is very similar with that of humans [28]. Thus, rabbits might be a good alternative model to investigate human BAT whitening. The interscapular regions are the major BAT depots of rabbits [27]. Our previous studies showed that the cell size of classical BAT increased during BAT whitening in rabbits and revealed this process was accompanied by the downregulation of BAT-selective genes (e.g., UCP1, PPARGC1A, and CYTB) and the upregulation of WAT-selective genes (e.g., LEP, SNCG, and CCDC80) and lipogenesis-related genes (e.g., FABP4 and GSN) using RT-qPCR and RNA-seq [29]. To investigate the ceRNA networks during BAT whitening, we performed miRNA-seq to identify the miRNA dynamics of BATs during BAT whitening in rabbits. Then circRNA-seq of a pooled sample was employed to assist whole-transcriptome sequencing datasets to identify circRNA dynamics of BATs. Furthermore, a combined analysis of miRNAs, circRNAs, and the retrieved lncRNAs and mRNAs data constructed the ceRNA networks during BAT whitening. Our work provides a catalog of miRNAs and circRNAs and the interaction networks of circRNAs, lncRNAs, miRNAs, and mRNAs involved in BAT whitening in rabbits, which may facilitate new insight into BAT-based obesity treatments.

## Methods

### Ethics approval

All experiments were performed in accordance with relevant guidelines and adher to the ARRIVE guidelines (https://arriveguidelines.org/) for the reporting of animal experiments. This study was carried out in accordance with the principles of the Basel Declaration and recommendations of the Guide for the Care and Use of Laboratory Animals (http://grants1.nih.gov/grants/olaw/references/phspol.htm). All surgical procedures involving rabbits were performed according to the approved protocols of the Biological Studies Animal Care and Use Committee, Sichuan Province, China. The protocol was approved by the ethics committee of Sichuan Agricultural University under permit No. DKY2020102011.

### Sample preparation

In this study, the Tianfu Black rabbits (native species in Sichuan province of China) were raised at the breeding center of Sichuan Agricultural University, Ya’an, China. These rabbits were fed a standard diet as described in our previous study and water ad libitum [16]. The rabbits were euthanized by air injection into the ear vein. BATs were collected from the interscapular regions of 12 male rabbits at four growth stages of day0 (D0), D15, D85, and 2 years (Y2) (three individuals per stage). These samples were isolated from the same rabbits subjected to our previous RNA-seq study, which revealed the downregulation of *UCP1*, *COX1*, and *PPARGC1A* and the upregulation of *ACSS2*, *ACLY*, *SNCG*, *LEP*, and *CCDC80* [29] (Figure S1A). On the other hand, a well reproducibility among replicates in corresponding group was also found in the previous RNA-seq data [29] (Figure S1B). For miRNA-seq and circRNA-seq, the adipose tissues were immediately isolated from rabbits under sterile conditions, snap-frozen in liquid nitrogen, and stored at -80 °C until RNA extraction.

### Immunofluorescence (IF)

For IF assay, specimens were incubated at 4 °C overnight in 4% paraformaldehyde, embedded in paraffin, and sliced in 5 μm thick using a microtome (Leica, Bensheim, Germany). Slices were incubated with UCP1 antibody (anti-UCP1 rabbit polyclonal antibody; 1:500; purchased from Sangon Biotech, Shanghai, China; register number: D262447) overnight. Then, these slices were washed three times using phosphate buffer saline (PBS), incubated in the fluorescein isothiocyanate-conjugated secondary antibody (1:500; BOSTER, Wuhan, China; register number: BM2012) for 1 hour at 37 °C, and incubated in DAPI for 5 minutes. After being washed three times using PBS, all slices of IF were imaged using an Olympus BX-50F light microscope (Olympus Optical, Tokyo, Japan).

### Sequencing and data analysis of miRNA

Total RNA was extracted using TRIzol Reagent (Life Technologies, Carlsbad, CA, USA) according to the manufacturer’s instructions. RNA quality check was performed using a Nanodrop2000 (Thermo Fisher Scientific, Waltham, MA, USA) and an Agilent 2100 Bioanalyzer (Agilent Technologies, CA, USA). The RNA samples with concentration > 200 ng/μL, absorbance at 260/280 nm (A260/A280) > 1.8 and < 2.2, A260/A230 ≥ 2.0, and RIN ≥ 7 were used for the library construction. The miRNA-seq library was constructed using TruSeq Small RNA Sample Prep Kits (Illumina, San Diego, USA) according to the manufacturer’s instructions. Briefly, after adding adapters at 3’ termination and 5’ termination, the miRNAs were reversely transcribed to 1st strand cDNA, and the cDNA was used for PCR amplification. The PCR product was purified using 6% polyacrylamide gel electrophoresis, and the insert size of the library was assessed using Agilent 2100 Bioanalyzer (Agilent Technologies, CA, USA). Finally, the qualified libraries were sequenced on an Illumina NovaSeq 6000 platform, and 50 bp single-end reads were obtained.

The adapter sequences and low-quality reads of miRNA-seq were removed using cutadapt (v3.2) [30] with parameters of ‘-e 0 -a AGATCGGAAGAGCACACGTCTGAACTCCAGTCAC -m 18 -M 30 -O 3’. The clean reads were then mapped to the latest miRbase [31] and Rfam database (v14.4) [32] using bowtie2 (v2.4.5) [33] to identify annotated miRNAs. The sequences that did not overlap with any annotated sequence were classified as unannotated reads. MirDeep2 (v2.0.1.2) [34] was used to identify novel miRNAs based on secondary structure, Dicer enzyme cleavage site, and minimum free energy indexes. The raw read counts estimated by MirDeep2 were used to analyze the differentially expressed miRNAs (DEmiRNAs) among four stages using DEseq2 (v1.34.0) [35]. The miRNAs with the thresholds of p-value < 0.05 and [log2(Fold-change) > 1 (log2FC >1) and < -1] were considered DEmiRNAs. To more accurately predict the miRNA target, we retrieved the mRNA expression data of these samples from our previous study [29]. The 3' UTR sequences of mRNAs were used for target prediction of miRNAs using miRanda software (v3.3a) [36] with default parameters, and the mRNAs that had an opposite expression with miRNAs (Spearman correlation coefficient < -0.6 with p-value < 0.05 across the 12 samples) was considered the potential target genes of miRNAs.

### Validation of miRNAs using RT-qPCR

The 5’ primers of miRNAs were designed based on the corresponding miRNA sequences (the U bases were replaced using T bases). Approximately 2 μg total RNA was reversed and transcribed to cDNA using Mir-X miRNA First-Strand Synthesis Kit (Takara, Dalian, China). The cDNA was then used as the template for PCR. The PCR was performed on a CFX96^TM^ Real-Time PCR Detection System (BioRad, California, USA) using the SYBR Premix Ex Taq^TM^ II (Novoprotein, Jiangsu, China). The PCR was conducted under the following conditions: pre-denaturation at 95 °C for 30 s, followed by 40 cycles of denaturation at 95 °C for 15 s and annealing/extension at 58.8 °C for 20 s. The Cq values of miRNAs were normalized to the *U6* gene using the 2^-ΔΔCt^ method. Three independent samples were set per stage (D0, D15, D85, and Y2), and two technical replicates were set for one individual sample.

### Identification of circRNAs from circRNA-seq and whole-transcriptome libraries

**Fig. 1:**
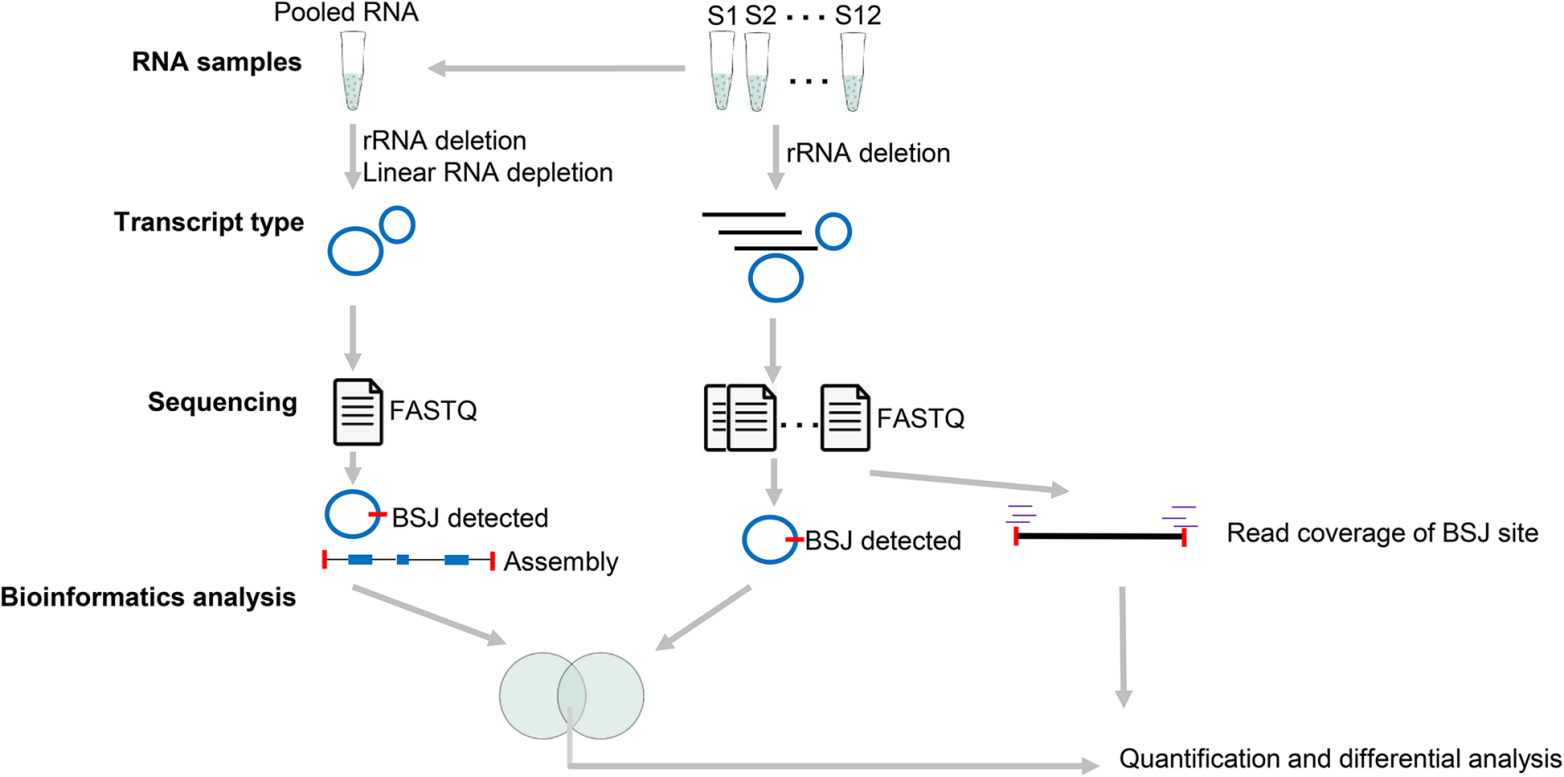
Identification and quantification of circRNA using circRNA-seq data and whole-transcriptome sequencing data. The transcripts marked by black lines and blue circles represent linear RNAs and circRNAs, respectively. The pooled RNA sample was used for the identification of circRNAs and the assembly of spliced circRNAs. The RNA samples in whole-transcriptome sequencing data were used to identify and quantify circRNA based on BSJ reads. The circRNAs identified by the two types of libraries were used for downstream analyses.

CircRNA can be identified using whole-transcriptome sequencing. The existence of both linear transcripts and circular transcripts was reported to increase the degree of difficulty in distinguishing sequencing reads from linear RNA and circRNA when performing transcript assembly of circRNAs [37]. To investigate the circRNA dynamics, circRNA-seq of pooled sample was employed to assist whole-transcriptome sequencing datasets to identify circRNAs of BATs (Fig. 1). Briefly, the RNA samples extracted from the 12 BATs were pooled as one sample, and then this RNA sample was used to construct a circRNA-seq library. The linear RNA of total RNA was digested to enrich circRNAs using RNase R (Epicentre, USA). The sequencing library was prepared using NEBNext® Ultra™ Directional RNA Library Prep Kit for Illumina® (NEB, USA) following the manufacturer’s recommendations. Then second-strand cDNA was generated using DNA Polymerase I and RNase H, during which dTTPs were replaced by dUTPs. After adding sequencing adapters, the segments with 250-300 bp length were purified using the AMPure XP system (Beckman Coulter, Beverly, USA). The purified segments underwent PCR amplification, product purification, and library quality check. Finally, the quantified library was sequenced on an Illumina novaseq-6000 platform, and 150 bp paired-end reads were generated. The low-quality reads and sequencing adapters were removed using Fastp software (v0.23.2) [38]. The clean reads were mapped to the rabbit genome (OryCun2.0, Ensembl release 101) using Hisat2 (v2.2.1) [39] with default parameters. The circRNA identification was conducted using Find_circ (v1.2) [40] and CIRI2 (v2.0) [41]. The 8147 circRNAs that were detected by both two types of software were used.

Although the whole-transcriptome sequencing library contains both linear RNA and circRNAs, which led us to access the internal structures of circRNAs hardly, it could provide reliable back-splicing information to identify circRNAs [40]. In this study, the datasets of whole-transcriptome sequencing of the 12 samples were retrieved from our previous study [16] and were subjected to identification and quantification of circRNAs based on the back-splicing junction (BSJ) reads. Briefly, the clean reads of whole-transcriptome sequencing were mapped back to the rabbit genome using the non-splicing mode of Bowtie2 software (v2.4.5) [33]. For the unmapped reads, the 20 bp sequence of 5’ termination and 20 bp sequence of 3’ termination of each read were extracted using Samtools (v1.15.1) [42] and used to distinguish the linear and circular splicing by remapping back to the rabbit genome using splicing-mode of Bowtie2 software (v2.4.5) [33]. The script “find_circ.py” in Find_circ (v1.2) [40] was then used to identify potential circRNAs. The potential circRNA with at least two unique BSJ reads with GT/AG splicing signals in one sample was used. Finally, the circRNAs identified in both circRNA-seq and whole-transcriptome were merged based on the BSJ sites, and circRNA determined by the two types of libraries were considered credible circRNAs. The raw counts of total reads that mapped to the circRNA BSJ sites were subjected to differential analysis using DEseq2 (v1.34.0) [35] (Fig. 1). The circRNAs with the thresholds of *p*-value < 0.05 and the | log2FC | >1 were considered differentially expressed circRNAs (DECs).

### Validation of circRNAs using divergent primers and Sanger sequencing

To validate the circRNAs identified in this study, we randomly selected nine circRNAs to verify their existence in our samples. The total RNA extracted from the 12 BAT samples was pooled, and linear RNA was digested using RNase R (Epicentre, USA). Then the remaining RNA was reversed and transcribed to complementary DNA (cDNA) using PrimeScripts RT Reagent Kit containing gDNA Eraser (TAKARA, Dalian, China). The genome DNA was extracted from the BAT of a rabbit using Animal Tissue DNA Isolation Kit (FOREGENE Biotech, China). For circRNA validation, we referenced a previous study by Memczak and colleagues [40]. A pair of divergent primers across the back-splicing junctions were designed to validate the circular sites in cDNA for each circRNA. The divergent primers were also used to amplify the gDNA to the correctness of primer designment. The products amplified from cDNA and gDNA using PCR were validated by 1.5% agarose gel electrophoresis. Furthermore, the PCR products with expected length sizes were retrieved using SanPre Column DNA gel Extraction Kit (Sangon Biotech, Shanghai, China) and subjected to Sanger sequencing to validate the back-splicing of RNA.

### Construction of circRNA-miRNA-mRNA and lncRNA-miRNA-mRNA triple networks

The lncRNA data of the 12 samples were retrieved from our previous studies [16]. The interaction between the head-to-tail sequences of circRNAs and miRNAs (or between lncRNAs and miRNAs) was predicted using miRanda software (v3.3a) with default parameters [36]. To predict the interaction between the circRNA BSJs and miRNAs, the tail 100 bp and head 100 bp sequences of circRNA were linked to be a short linear sequence and were then subjected to miRanda prediction with miRNAs [36]. The predicted binding sites of miRNAs that were across BSJs were used. Furthermore, the circRNA or lncRNA that had an opposite expression with miRNAs (Spearman correlation coefficient < -0.6 and p-value < 0.05 across the 12 samples) were used to construct the ceRNA network. The miRNAs predicted to interact with both circRNA and mRNA were used to construct the circRNA-miRNA-mRNA triple network. The miRNA predicted to interact with both lncRNA and mRNA were used to construct the lncRNA-miRNA-mRNA triple network. The lncRNA and circRNA sequences were mapped to NONCODEv6 database [43] and circBase [44] to predict the lncRNA and circRNA conservation between rabbits and humans using BLASTN (v2.13.0+) [45], respectively. The standard for sequence similarity was adopted from a previous similar study and was defined with a mapping identity of ≥70% and a total sequence identity of ≥75% in a covered region ≥100 nt [46].

### Functional annotation and pathway analysis

Gene Ontology (GO) enrichment and Kyoto Encyclopedia of Genes and Genomes (KEGG) pathway analysis were performed using the Database for Annotation, Visualization and Integrated Discovery (DAVID) [47, 48]. The enriched GO terms or KEGG pathways with a p-value < 0.05 were considered significant.

### Dual-luciferase reporter assays

The dual-luciferase reporter assays were performed using Duo-LiteTM Luciferase Assay System (Vazyme biotech, Nanjing, China). Briefly, the sequence containing the predicted miRNA interacting site was cloned into NheI-SalI site of pmirGLO vector. The 293T cells were seeded into 96-well plates. When the cell density reached 70–80%, the pmirGLO that had been cloned with wild type (wt) or mutant type (mu) was co-transfected with the synthetic ocu-miR-378-5p mimic or negative control (NC) into 293T cells using Lipofectamine® 2000 Reagent (Invitrogen, Carlsbad, CA, USA). The firefly luciferase activity was measured 48 h after transfection and was normalized to renilla luciferase activity.

### Statistical analysis

Statistical analyses, including T-test and One-way ANOVA were conducted on R software. The p-value < 0.05 was considered significant. The “*” represent “p-value < 0.05” in a statistical test and the “**” represent “p-value < 0.01” in a statistical test.

### Availability of data

The original data files have been uploaded and published to the NCBI SRA database. The accession number is PRJNA716375 (https://www.ncbi.nlm.nih.gov/bioproject/?term=PRJNA716375, SRA accession numbers were SRS8536080 - SRS8536083, SRS8536092, and SRS8536097- SRS8536103) and PRJNA854761 (https://www.ncbi.nlm.nih.gov/bioproject/PRJNA854761, SRA accession numbers were: SRS13642706 - SRS13642718).

## Results

### Identification of miRNAs during BAT whitening in rabbits

**Fig. 2:**
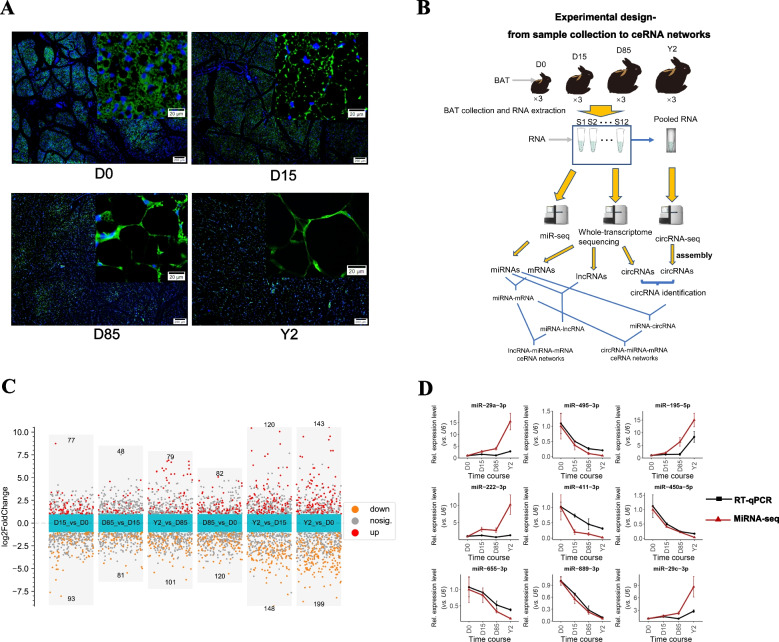
Immunofluorescence (IF) assay and miRNA analysis of rabbit BATs at four growth stages. **A** IF of BATs at four stages. Two different magnifications are presented in the IF assay (200 μm and 20 μm). The nucleus were stained using DAPI. The blue and green signals represent the nucleus and UCP1 proteins, respectively. **B** Construction of circRNA- and lncRNA-related ceRNA networks during BAT whitening in rabbits. **C** Differential analysis of miRNAs. The scatter plot showed miRNAs with log2FC > 1 and < -1 in the six comparisons. The red and orange points show the miRNAs with a p-value < 0.05. **D** Validation of nine differentially expressed miRNAs (DEmiRNAs) using RT-qPCR. The red and black lines represent the miRNA-seq and RT-qPCR, respectively. The expression was normalized to the *U6* gene and D0. The data shows the means of three independent experiments. Two technical replicates were set for one individual experimental replicate

The BATs were isolated from rabbits at stages of 0 days (D0), D15, D85, and 2 years (Y2) (n = 3 per group). The immunofluorescence (IF) assay showed that the interstitial spaces and the content of UCP1 protein were decreased from D0 to Y2. On the other hand, the samples in D85 showed high heterogeneity in UCP1+ cells (Fig. 2A). To comprehensively investigate the ceRNA networks involved in BAT whitening in rabbits, we collected BAT samples from D0, D15, D85, and Y2 rabbits and performed miRNA-seq and circRNA-seq. By combining our previously published whole-transcriptome data (from the same samples) [16], we set up to construct circRNA- and lncRNA-involved ceRNA networks during BAT whitening in rabbits, respectively (Fig. 2B).

MiRNA-seq data from the BATs provided an average of 14.54 million clean reads per sample after quality control. The sequencing base Q30 values ranged from 95.70% to 98.00% (Table S1). The distribution of length size showed that most of the clean reads were 21-24 bp, and the reads with a length size of 22 bp were the most abundant, indicating the well-constructed miRNA-seq libraries (Figure S2A). In total, 516 known miRNAs and 671 novel miRNAs were identified in our miRNA-seq libraries. Quantifying miRNA expression found that a total of 870 miRNAs (437 known and 433 novel miRNAs) were expressed in all four detected stages (Figure S2B). The pairwise comparisons of different growth stages identified 77, 48, 79, 82, 120, and 143 miRNAs were significantly upregulated in D15 *vs.* D0, D85 *vs.* D15, Y2 *vs.* D85, D85 *vs.* D0, Y2 *vs.* D15, and Y2 *vs.* D0, respectively (log2FC > 1 and p-value < 0.05). On the other hand, 93, 81, 101, 120, 148, and 199 miRNAs were significantly downregulated in D15 *vs.* D0, D85 *vs.* D15, Y2 *vs.* D85, D85 *vs.* D0, Y2 *vs.* D15, and Y2 *vs.* D0, respectively (log2FC < -1 and *p*-value < 0.05, Fig. 2C). The RT-qPCR detected the expression patterns of nine randomly selected known differentially expressed miRNAs (DEmiRNAs), including ocu-miR-29a-3p, ocu-miR-495-3p, ocu-miR-195-5p, ocu-miR-222-3p, ocu-miR-411-3p, ocu-miR-450a-5p, ocu-miR-655-3p, ocu-miR-889-3p, and ocu-miR-29c-3p. Our results showed that the vast majority (7 of 9) of these miRNAs demonstrated similar expression with miRNA-seq (Fig. 2D and Table S2), indicating the credible miRNA-seq data.

### Dynamics of miRNAs during the BAT whitening

**Fig. 3:**
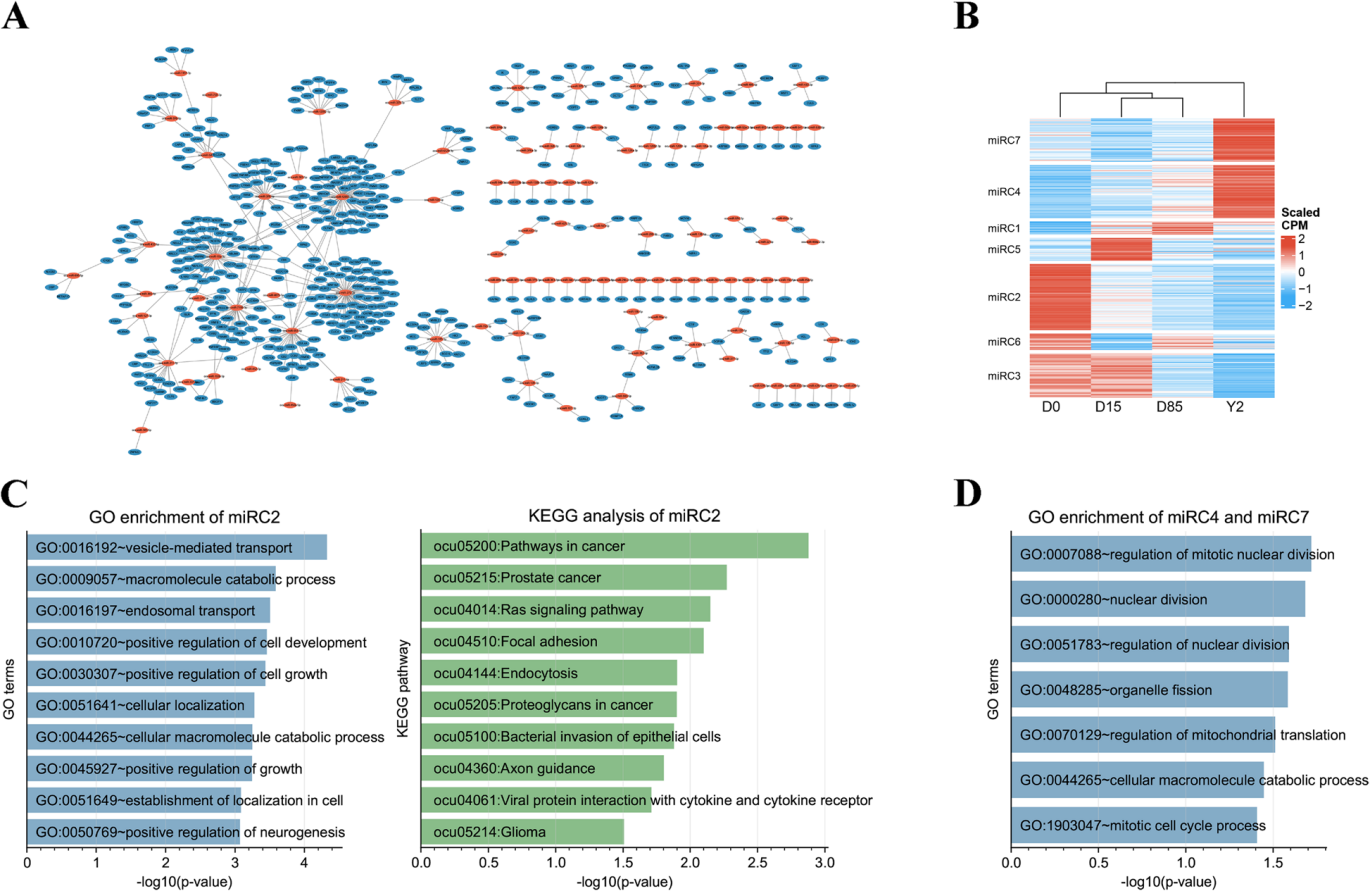
Target prediction and functional annotation of miRNAs. **A** Target genes of DEmiRNAs. The red and blue nodes show the miRNAs and mRNAs, respectively. Each edge shows the targeting relationship from miRNAs to mRNAs. **B** K-means clustering of DEmiRNAs based on expression levels. **C** Gene Ontology in biological process (GO-BP) enrichment and KEGG pathway analysis of target genes of miRNAs in miRC2. **D** GO-BP enrichment for the target genes of miRC4 and miRC7

The union set of known DEmiRNAs in all pairwise comparisons, including upregulated and downregulated ones, were subjected to the downstream target prediction. A total of 103 out of 298 known DEmiRNAs were predicted to target 484 mRNAs, and 555 miRNA-mRNA pairs were identified, of which miRNAs and mRNAs had opposite expression patterns across the 12 samples (Spearman correlation coefficient < -0.6 with p-value < 0.05) (Fig. 3A). The top 5 miRNAs that have the most number of target genes were ocu-miR-370-3p (71 targets), ocu-miR-12093-3p (67 targets), ocu-miR-18a-3p (49 targets), ocu-miR-7180-3p (36), and ocu-miR-378-5p (29 targets). The expression of all the five miRNAs showed highly expressed at D0 and then dramatically downregulated at D15 (Figure S3A). K-means clustering sorted all DEmiRNAs into 7 clusters (miRC1-miRC7, Fig. 3B). The miRNAs in miRC2 (76 miRNAs) represent the D0-selective miRNAs, which were highly expressed in D0 and low expressed in D15, D85, and Y2. A total of 47 miRNAs in miRC2 were predicted to target 351 mRNAs (Figure S3B). Functional annotation showed that a total of 188 Gene Ontology in biological process (GO-BP) terms were significantly enriched by the miRC2. The top 3 significantly enriched GO-BP terms by the target genes of miRC2 were vesicle-mediated transport, macromolecule catabolic process, and endosomal transport (Fig. 3C). On the other hand, immune response-associated GO-BP terms were also significantly enriched by miRC2, such as immune response-activating signal transduction, immune response-regulating signaling pathway, and activation of immune response (Table S3). A total of 16 KEGG pathways were significantly enriched by the miRC2. The top 3 enriched KEGG pathways were pathways in cancer, prostate cancer, and Ras signaling pathway (Fig. 3C). The MAPK signaling pathway and PI3K-Akt signaling pathway were also significantly enriched by miRC2 (Table S4). The miRNAs in miRC5 (26 miRNAs) and miRC1 (15 miRNAs) represent the D15-selective and D85- selective miRNAs, respectively. No target genes were predicted by the miRNAs in the two clusters. The miRNAs in miRC4 (61 miRNAs) and miRC7 (50 miRNAs) represent the Y2-selective miRNAs. A total of 10 miRNAs in miRC4 were predicted to target 9 mRNAs (Figure S3C), and 11 miRNAs in miRC7 were predicted to target 36 mRNAs (Figure S3D). A total of 7 GO-BP terms were significantly enriched by the two clusters. The top 3 enriched GO-BP terms were regulation of mitotic nuclear division, nuclear division, and regulation of nuclear division (Fig. 3D). There were no KEGG pathways that were significantly enriched by the miRC4 and miRC7.

### Genome-wide identification of circRNAs using circRNA-seq and whole-transcriptome sequencing data

**Fig. 4:**
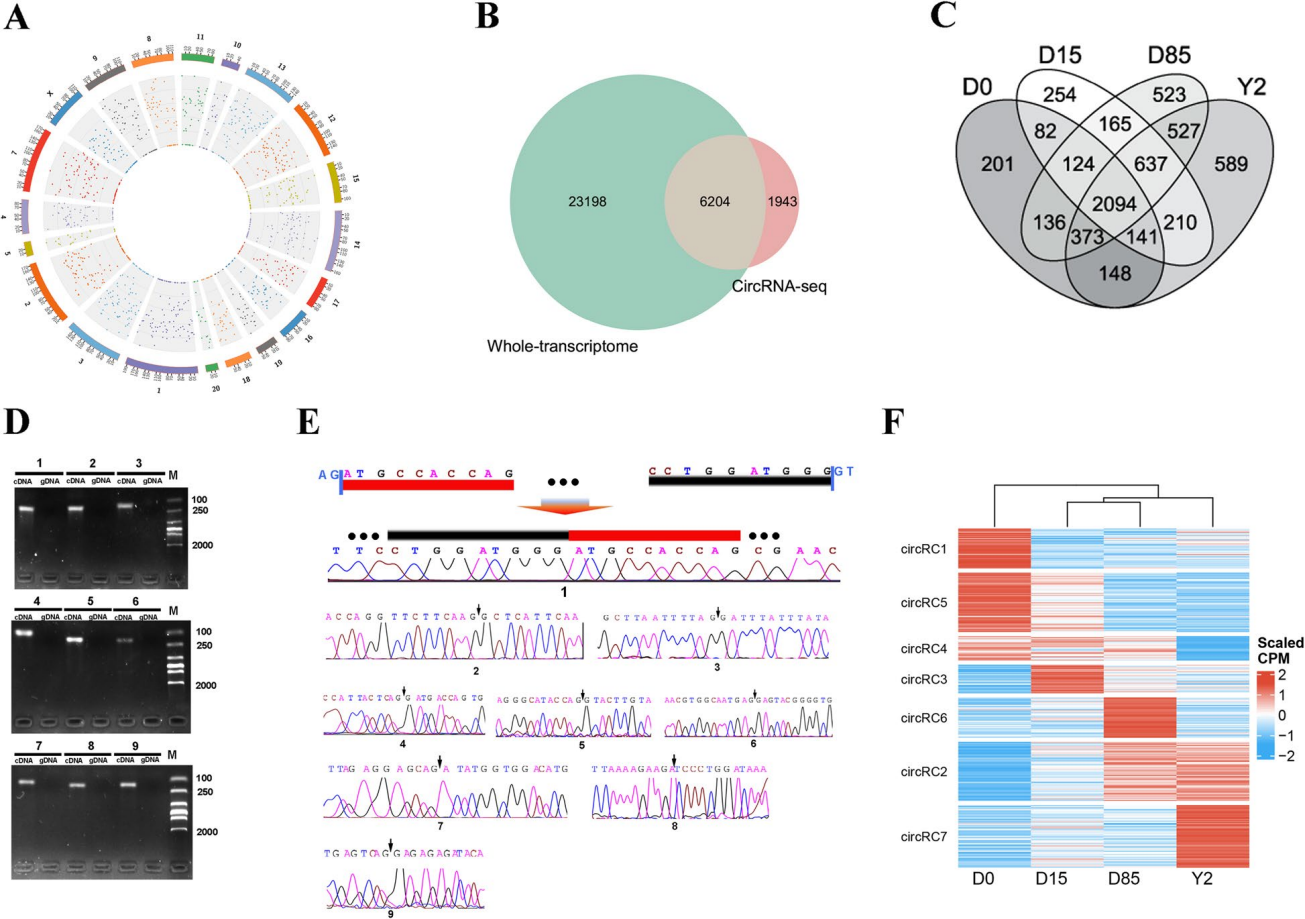
Genome-wide identification and differential analysis of circular RNAs (circRNAs) of BATs in rabbits. **A** Genomic distribution of circRNAs in a pooled RNA sample using circRNA-seq. **B** Venn diagram analysis of the circRNAs identified from circRNA-seq and whole-transcriptome data. **C** The Venn diagram analysis of expressed circRNAs among different growth stages. **D** Divergent primers amplify circRNAs. The IDs of circRNA were listed in Table S6. “M” represents “DNA marker”. The bands with expected length size were extracted and subjected to Sanger sequencing. The gels were cropped from Figure S5. **E** Sanger sequencing confirms head-to-tail splicing. The splicing mode and the GT/AG splicing signal of one circRNA were shown. The black arrows are pointing the back-splicing sites of the other eight circRNAs. **F** K-means clustering of DECs based on expression levels.

To identify genome-wide circRNAs and obtain a set of well-assembled circRNAs, we first performed circRNA-seq for a pooled RNA sample from the 12 BATs. A total of 105.10 million clean reads were obtained from the sample, with a Q30 of 91.85% (Table S1). In total, 8147 circRNAs were identified in the library, distributed throughout the rabbit genome (Fig. 4A). Most circRNAs identified in the circRNA-seq library contained 2-5 exons, had 200-600 bp spliced lengths, and were annotated into exons of protein-coding genes (Figure S4A). Analysis of the whole-transcriptome sequencing data from the 12 individual samples in our previous study [16] found a substantial proportion of linear splicing events and a small proportion of back-splicing events (Figure S4B). A total of 29402 circRNAs with support from at least two independent back-splicing junction (BSJ) reads were identified in the whole-transcriptome sequencing data. Based on the BSJ information, we compared circRNAs in the circRNA-seq data and the whole-transcriptome sequencing data and identified 6204 credible circRNAs (Table S5, Fig. 4B), among which 2094 circRNA were expressed in all four detected stages of BAT whitening (Fig. 4C). The circRNAs were validated by the RT-PCR assay that amplified across BSJs. The production of cDNA amplified by the divergent primers of nine randomly selected circRNAs showed their bands at the corresponding positions, while that of genomic DNA (gDNA) amplified by the divergent primers showed no bands (Fig. 4D and Figure S5) (the primers and product length sizes were shown in Table S6). Furthermore, the Sanger sequencing of the PCR products of the nine circRNAs showed the RNA back-splicing of transcriptions (Fig. 4E), indicating credible circRNA data.

When comparing samples by groups, a total of 38, 5, 31, 74, 15, and 87 circRNAs were significantly upregulated in D15 *vs.* D0, D85 *vs.* D15, Y2 *vs.* D85, D85 *vs.* D0, Y2 *vs.* D15, and Y2 *vs.* D0, respectively. A total of 26, 4, 27, 54, 17, and 71 circRNAs were significantly downregulated in D15 *vs.* D0, D85 *vs.* D15, Y2 *vs.* D85, D85 *vs.* D0, Y2 *vs.* D15, and Y2 *vs.* D0, respectively. (Figure S6A). K-means clustering sorted all differentially expressed circRNAs (DECs) into 7 clusters (circRC1-circRC7, Fig. 4F). The circRNAs in circRC1 (38 circRNAs) and circRC5 (56 circRNAs) represent the D0-selective circRNAs, which were highly expressed in D0 and low expressed in D15, D85, and Y2. Analysis of the host genes of circRC1 and circRC5 found that 7 GO-BP terms were significantly enriched, of which protein modification process, cellular protein modification process, and mitotic nuclear division were the top 3 enriched GO-BP terms (Figure S6B). KEGG pathway analysis showed that 6 KEGG pathways were significantly enriched, of which transcriptional misregulation in cancer, adherens junction, and longevity regulating pathway were the top 3 enriched pathways (Figure S6C). The circRNAs in circRC3 (27 circRNAs) and circRC6 (38 circRNAs) represent the D15-selective and D85-selective circRNAs, respectively. No GO-BP terms and KEGG pathways were enriched by the host genes of circRNAs in the two clusters. The circRNAs in circRC7 (59 circRNAs) represent the Y2-selective circRNAs. Analysis of the host genes of circRC7 found that 18 GO-BP terms were significantly enriched, of which cellular component assembly, cellular component biogenesis, and regulation of lipid metabolic process were the top 3 enriched GO-BP terms (Figure S6B). KEGG pathway analysis showed that the FoxO signaling pathway and ubiquitin mediated proteolysis were significantly enriched by the host genes of circRNAs in circRC7 (Figure S6C).

### Construction and functional annotation of circRNA-related ceRNA networks

**Fig. 5:**
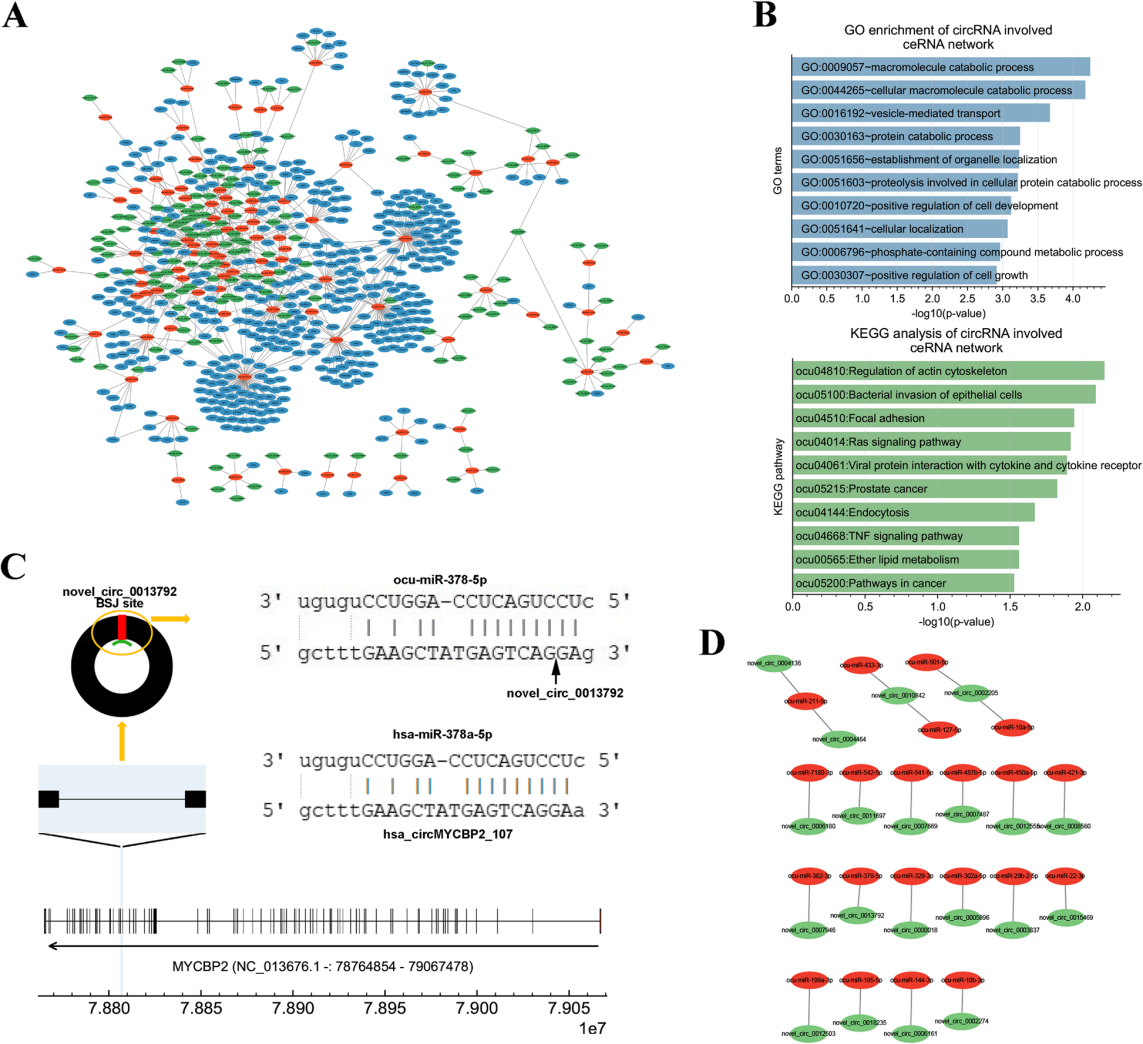
Construction of circRNA-related ceRNA networks. **A** Construction of circRNA-related ceRNA network based on the head-to-tail linear sequences of circRNAs. The red, blue, and green nodes show the miRNAs, mRNAs, and circRNAs, respectively. **B** GO-BP enrichment and KEGG pathway analysis of circRNA-related ceRNA network. **C** An example of miRNAs interacting with the BSJ site of circRNAs. The black line shows the exons of *MYCBP2*. The 64th and 65th exon of *MYCBP2* were highlighted and zoomed in a new window with a blue shadow. The predicted interaction between novel_circ_0013792 and ocu-miR-378-5p is shown in a yellow circle. The red block shows the BSJ site of circRNA. In the top-right panel, the interaction between miRNA and circRNA was shown in single base resolution. The black arrow points to the BSJ site of circRNA, which was located in the interaction region between miRNA and circRNA. The interaction between human hsa-circMYCBP2_107 and hsa-miR-378-5p is shown. **D** Prediction of the novel interaction pairs between circRNA BSJ sites and miRNAs

Prediction of the head-to-tail linear sequences of circRNAs found that 275 circRNA interacted with 245 DEmiRNAs, and 778 circRNA-miRNA interaction pairs were identified. We next extracted miRNAs paired with both circRNAs and mRNAs to construct the circRNA-miRNA-mRNA triple networks, which included 155 circRNAs, 90 miRNAs, 462 mRNAs, and 1979 circRNA-miRNA-mRNA regulatory axes (Fig. 5A and Table S7). Analysis of the sequences of circRNAs in the networks found that 130 circRNAs were conserved between rabbits and humans (Table S7). GO analysis showed that 169 GO-BP terms were significantly enriched by the network. The top 3 enriched GO-BP terms were macromolecule catabolic process, cellular macromolecule catabolic process, and vesicle-mediated transport (Fig. 5B). In addition, the immune response-associated GO-BP terms were also enriched by the network (Table S9). KEGG pathway analysis showed that 13 signal pathways were significantly enriched by the network. The top 3 enriched KEGG pathways were regulation of actin cytoskeleton, bacterial invasion of epithelial cells, and focal adhesion (Fig. 5B).

Based on the expression patterns of circRNAs in Fig. 4G, we analyzed stage-selective circRNAs in circRNA-miRNA-mRNA networks. Our data showed that no D0-selective circRNA in circRC1 was predicted to function in circRNA-miRNA-mRNA networks, and 9 circRNA in circRC5 were predicted to regulate 9 mRNAs via interacting with 8 miRNAs (e.g., novel-circ-0014809 - ocu-miR-27b-3p - *SLC22A3* regulatory axis) (Figure S7A). For the D15-selective circRNAs (circRC3), 4 circRNAs were predicted to regulate 13 mRNA via interacting with 5 miRNAs (e.g., novel-circ-0005656 - ocu-miR-874-3p - *IDH2* regulatory axis) (Figure S7B). For the D85-selective circRNAs (circRC6), 3 circRNAs were predicted to regulate 8 mRNA via interacting with 3 miRNAs (e.g., novel-circ-0013332 - ocu-miR-433-3p - *CSF1* regulatory axis) (Figure S7C). Because of the small number of mRNAs regulated by D0-, D15-, and D85-selective circRNAs, no significant GO terms and KEGG pathways were enriched. For the Y2-selective circRNAs (circRC7), 22 circRNAs were predicted to regulate 352 mRNA via interacting with 42 miRNAs (Figure S7D). The top 3 significantly enriched GO-BP terms by the Y2-selective circRNAs involved ceRNA network were vesicle-mediated transport, peptidyl-serine modification, and phosphate-containing compound metabolic process (Figure S7E). The top 3 significantly enriched KEGG pathways by the Y2-selective circRNA-related ceRNA network were pathways in cancer, bacterial invasion of epithelial cells, and endocytosis (Figure S7E). On the other hand, the MAPK signaling pathway was also significantly enriched by the Y2-selective circRNAs involved ceRNA network.

Theoretically, RNA cyclization could provide new MREs in the BSJ sites of circRNAs. For instance, *MYCBP2* is a protein-coding gene containing 84 exons at chromosome 8. The back-splicing of the 64th and 65th exon formed novel_circ_0013792. The head-to-tail linear sequences of novel_circ_0013792 have no MRE for ocu-miR-378a-5p, a master thermogenic miRNA of BAT [49], which downregulated during BAT whitening in this study. The RNA cyclization of novel_circ_0013792 formed an ocu-miR-378-5p MRE at its BSJ (Fig. 5C). Both the novel_circ_0013792 (corresponding to hsa-circMYCBP2_107 in human) and ocu-miR-378-5p (corresponding to hsa-miR-378a-5p in human) was conserved between humans and rabbits. Analysis of BSJ sequences of all circRNAs found that there were 22 novel circRNA-miRNA pairs that were not found in the prediction performed using the head-to-tail linear sequences of circRNAs (Fig. 5D). Some of the miRNAs in these interaction pairs were reported to be involved in the thermogenesis of adipocytes, such as from D0 to D15 dramatically downregulated ocu-miR-378-5p (log2FC = -2.56 in Y2 *vs.* D0) [49] and ocu-miR-433-3p (log2FC = -5.39 in Y2 *vs.* D0) [50] and from D15 to D85 dramatically upregulated ocu-miR-22-3p (log2FC = 1.69 in Y2 *vs.* D0) [51].

### Construction and functional annotation of lncRNA-related ceRNA networks

**Fig. 6:**
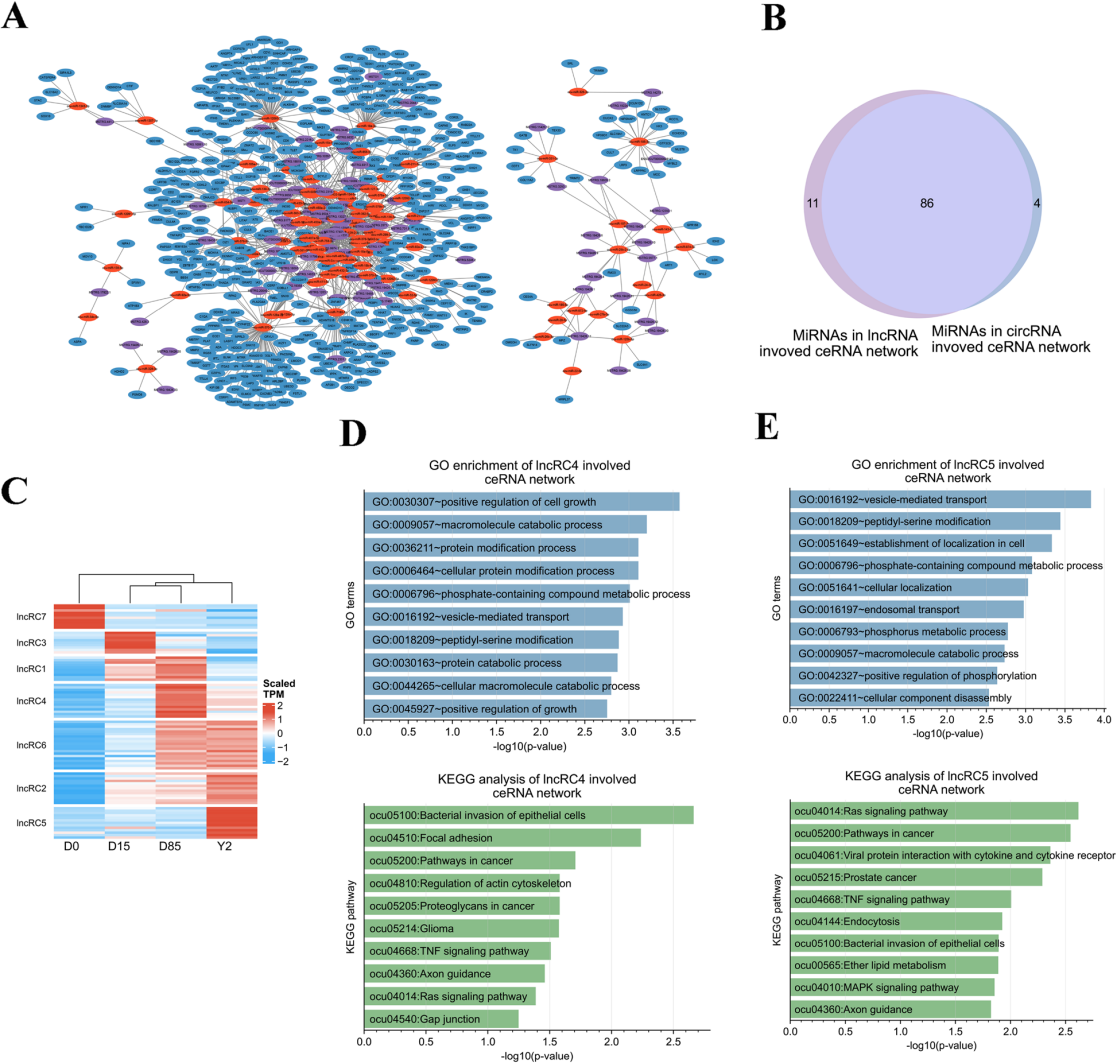
Construction of lncRNA-related ceRNA networks. **A** Construction of lncRNA-related ceRNA network. The red, blue, and purple nodes show the miRNAs, mRNAs, and lncRNAs, respectively. **B** Comparing miRNAs involved in lncRNA-miRNA-mRNA networks and circRNA-miRNA-mRNA networks. **C** K-means clustering of lncRNAs based on expression levels. **D** GO-BP enrichment and KEGG pathway analysis of D85-selective lncRNA-related ceRNA network. **E** GO-BP enrichment and KEGG pathway analysis of Y2-selective lncRNA-related ceRNA network

Prediction of the lncRNA data found that 112 lncRNAs interacted with 223 DEmiRNAs and 998 lncRNA-miRNA interaction pairs were identified. We next extracted miRNAs paired with both lncRNAs and mRNAs to construct the lncRNA-miRNA-mRNA triple networks, which included 89 lncRNAs, 97 miRNAs, 477 mRNAs, and 5064 lncRNA-miRNA-mRNA regulation axes (Fig. 6A and Table S8). Analysis of the sequences of lncRNAs in the networks found that 43 lncRNAs were conserved between rabbits and humans (Table S8). Interestingly, a great portion of miRNAs in the lncRNA-miRNA-mRNA triple networks (88.66%) overlapped the miRNAs in the circRNA-miRNA-mRNA triple network. As expected, the lncRNA-miRNA-mRNA networks were involved in similar GO-BP terms and KEGG pathways enriched by the circRNA-miRNA-mRNA network. On the other hand, there were 11 (ocu-miR-12091-5p, ocu-miR-12092-3p, ocu-miR-128a-3p, ocu-miR-128b-3p, ocu-miR-1307-3p, ocu-miR-18a-5p, ocu-miR-24-3p, ocu-miR-28-5p, ocu-miR-32-5p, ocu-miR-409-5p, ocu-miR-6529-3p) and 4 miRNAs (ocu-miR-140-3p, ocu-miR-184-3p, ocu-miR-25-3p, ocu-miR-574-3p) were predicted to specifically interact with lncRNAs and circRNAs, respectively (Fig. 6B).

K-means clustering sorted the lncRNAs in the lncRNA-miRNA-mRNA triple networks into 7 clusters (lncRC1-lncRC7, Fig. 6C). We then analyzed stage-selective lncRNAs in lncRNA-miRNA-mRNA networks. Our data showed that 10 D0-selective lncRNAs (lncRC7) were predicted to regulate 9 mRNAs via interacting with 9 miRNAs (Figure S8A). For the D15-selective lncRNAs (lncRC3), 9 lncRNAs were predicted to regulate 38 mRNAs via interacting with 10 miRNAs (Figure S8B). Because of the small number of mRNAs regulated by D0- and D15-selective lncRNAs, no significant GO terms and KEGG pathways were enriched. For the D85-selective lncRNAs (lncRC4), 14 lncRNAs were predicted to regulate 371 mRNAs via interacting with 55 miRNAs (Figure S8C). The top 3 significantly enriched GO-BP terms by the D85-selective lncRNA-related ceRNA network were positive regulation of cell growth, macromolecule catabolic process, and protein modification process (Fig. 6D). The top 3 significantly enriched KEGG pathways by the D85-selective lncRNA-related ceRNA network were bacterial invasion of epithelial cells, focal adhesion, and pathways in cancer (Fig. 6D). For the Y2-selective lncRNAs (lncRC5), 15 lncRNAs were predicted to regulate 374 mRNAs via interacting with 54 miRNAs (Figure S8D). The top 3 significantly enriched GO-BP terms by the Y2-selective lncRNA-related ceRNA network were vesicle-mediated transport, peptidyl-serine modification, and establishment of localization in cell (Fig. 6E); The top 3 significantly enriched KEGG pathways by the Y2-selective lncRNA-related ceRNA network were Ras signaling pathway, pathways in cancer, and viral protein interaction with cytokine and cytokine receptor (Fig. 6E).

### Validation of interaction pairs of ceRNA networks

**Fig. 7:**
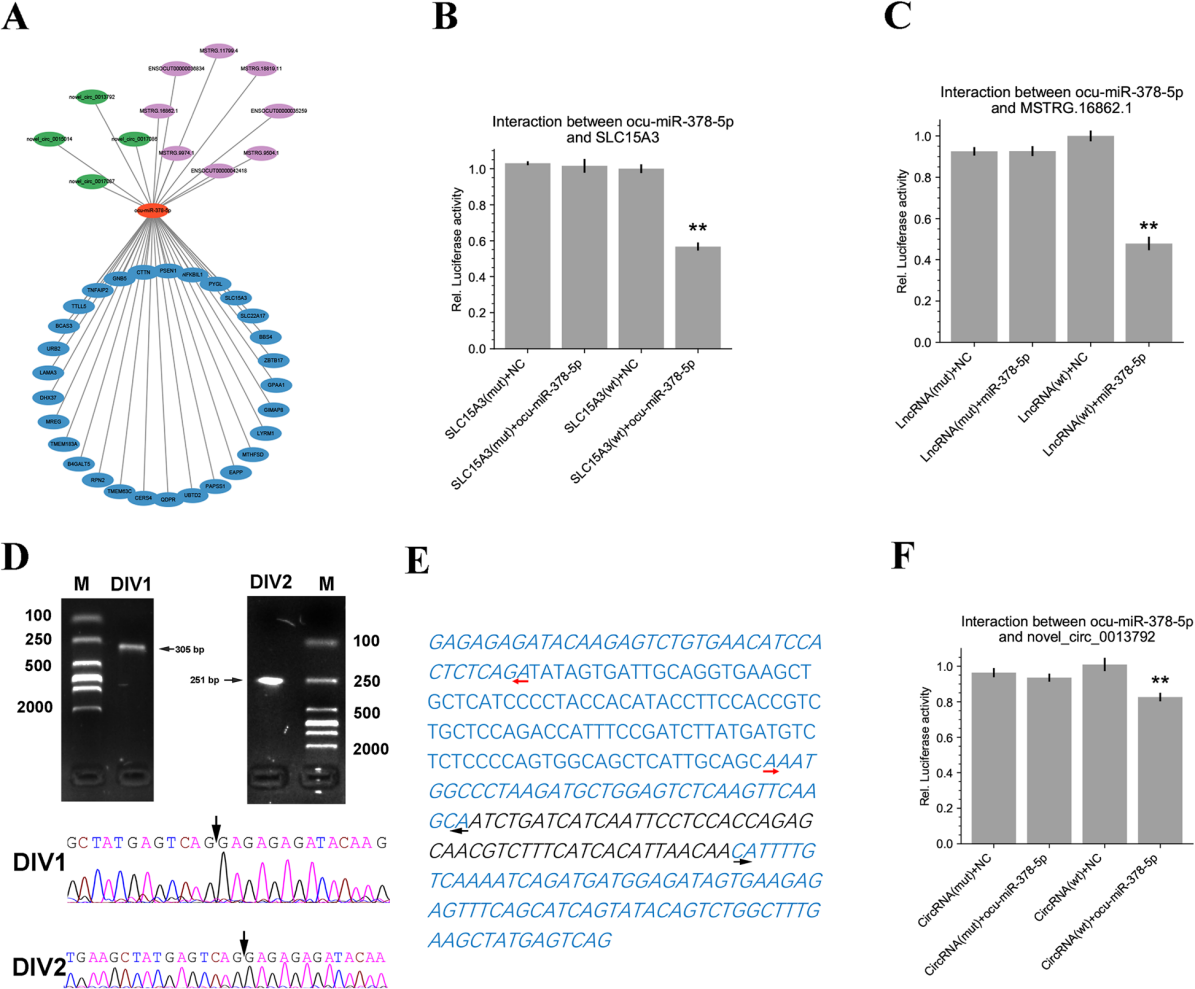
Validation of ocu-miR-378-5p-related ceRNA network. **A** Ocu-miR-378-5p-related ceRNA network. The green, purple, red, and blue nodes represent circRNA, lncRNA, miRNA, and mRNAs. **B** Dual-luciferase reporter assay validates the interaction between ocu-miR-378-5p and *SLC15A3*. The data in the dual-luciferase reporter assay shows the means of three independent experiments. The error bars show the standard errors of means. **C** Dual-luciferase reporter assay validates the interaction between ocu-miR-378-5p and MSTRG.16862.1. The data in the dual-luciferase reporter assay shows the means of three independent experiments. The error bars show the standard errors of means. **D** Validation of full length of novel_circ_0013792 by Sanger sequencing. “M” represents “DNA marker”. The bands with expected length size were extracted and subjected to Sanger sequencing. The gels were cropped according to the red lines in Figure S9. The black arrows point the BSJ sites of novel_circ_0013792 in Sanger sequencing. **E** Assembly of the full length of novel_circ_0013792. The blue and italicized bases show the sequence amplified by DIV1 primers and DIV2 primers, respectively. The black arrows show the binding sites and amplification directions of DIV1 primers. The red arrows show the binding sites and amplification directions of DIV2 primers (F) Dual-luciferase reporter assay validates the interaction between ocu-miR-378-5p and novel_circ_0013792. The data in the luciferase reporter assay shows the means of six independent experiments. The error bars show the standard errors of means

In this study, the D0-selective ocu-miR-378-5p, a well-known thermogenic miRNA in previous studies, showed the highest expression level among the top 5 miRNAs with the most target genes in this study (Table S2). The *SLC15A3* was targeted by ocu-miR-378-5p and was predicted to be involved in the immune response during BAT whitening (Table S3). Analysis of ocu-miR-378-5p found it interacted with 4 circRNAs and 8 lncRNAs (Fig. 7A), of which lncRNA MSTRG.16862.1 and the circRNA novel_circ_0013792 showed the highest “score” in the miRanda prediction. To validate the interactions between ocu-miR-378-5p and the other three types of RNA, we performed a dual-luciferase reporter assay for each interaction pair. Our result showed that miR-378-5p significantly reduced the luciferase activity via binding to the sequence of wild-type (wt) of *SLC15A3* and MSTRG.16862.1, while no significant reduction was found with their mutant (mu) sequences (Fig. 7B and C), confirming ocu-miR-378-5p directly targeting to *SLC15A3* and interacting with MSTRG.16862.1. Since the predicted binding site of miRNA in novel_circ_0013792 was located in the BSJ site of novel_circ_0013792 (its back-splicing had been validated in Fig. 4D), we first validated its full length to confirm that there were no other ocu-miR-378-5p binding sites by using two pair of divergent primers. Sanger sequencing of the extracted two bands from gels with expected sizes showed RNA back-splicing of transcription (Fig. 7D and Figure S9). The assembled circRNA by the two amplified sequences was fully identical to the predicted 318 bp circRNA sequence in the circRNA bioinformatics analysis, confirming only one binding site of ocu-miR-378-5p in the BSJ sites (Fig. 7E). The dual-luciferase reporter assay showed that ocu-miR-378-5p significantly reduced the luciferase activity via binding to the sequence of wild-type (wt) BSJ sequence of novel_circ_0013792, while no significant reduction was found with its mutant (mu) sequence (Fig. 7F), indicated that miR-378-5p directly interacted with BSJ site of novel_circ_0013792.

## Discussion

The UCP1 is a key marker for classical BAT. In this study, the expression levels of UCP1 protein decreased from D0 to Y2 in the IF assay, which was in line with the previous study and indicated BAT whitening in rabbits [16, 52, 53]. The adipose tissue is as an extraordinarily flexible and heterogeneous organ [54]. Interestingly, our result showed that the D85 samples are highly heterogeneous tissues in UCP1 expression, indicating a complex process of BAT whitening, and further single-cell technology may help to deconstruct the details of BAT whitening. In recent years, several miRNAs have been validated to play important roles in the thermogenesis of adipose tissues [55]. Our data showed that D0-selective miRNAs contained a well-known conserved thermogenic miRNA, ocu-miR-378-5p, indicating that the ocu-miR-378-5p and the other D0-selective miRNAs might play roles in maintaining the thermogenic phenotype of BATs. The previous study revealed that BAT played a role in mitigating the metabolic syndrome through BAT-derived exosomes, a type of vesicle that deliver functional molecules [56]. The D0-selective miRNAs were significantly enriched in vesicle-mediated transport, which suggested that miRNAs might be transported and exert their functions extracellularly. WAT had been reported to be associated with immune responses [57]. Previous studies have revealed that BAT whitening enables BAT to gain WAT properties, leading to adipose tissue inflammation in mice [58]. Our result showed that the targets of D0-selective miRNAs were involved in immune response-related biological processes. Since miRNAs generally inhibit the expression of target genes, the downregulation of D0-selective miRNAs might indicate the activation of the immune-related gene program during the early whitening process of BATs in rabbits. Except for the loss of thermogenic phenotype, lipid accumulation is another obvious characteristic of BAT whitening. The target genes of D0-selective miRNAs were significantly enriched in adipose development-related signal pathways, such as the Ras signaling pathway [59], MAPK signaling pathway [60], and PI3K-Akt signaling pathway [61], which suggested that the disinhibition of the target genes of D0-selective miRNAs in these signal pathways might promote adipogenesis during BAT whitening. The target genes of Y2-selective miRNAs were significantly enriched in cell proliferation-related biological processes, which might suggest that high expression of Y2-selective miRNAs could repress the adipocyte proliferation of BAT in old rabbits. The post-transcriptional repression through the miRNA seed region binding to 3' UTR of target mRNA is considered the canonical mode of miRNA-mediated gene regulation [62]. While our results showed that no target genes of the D15-selective and D85-selective miRNAs were predicted by the canonical theory. Here, we considered several possible reasons for this observation: (1) the incomplete annotation of the 3’ UTR sequence in current databases [63], (2) the target genes might tend to be regulated by other epigenetic factors, such as DNA methylation, chromatin accessibility, or histone modification [64], and/or (3) these miRNAs might regulate gene expression by other modes, such as miRNA regulating gene expression by binding to ORF regions [65].

Previous studies have identified circRNAs are a class of widely expressed ncRNAs [40, 66]. In rabbits, circRNAs have been annotated in muscle [67], carotid arteries [68], and embryo [69]. However, no adipose circRNA data were annotated in rabbits. As expected, our whole-transcriptome data showed a substantial proportion of linear splicing events while only a small proportion of back-splicing events, indicating potential difficulty in circRNA assembly. To address this issue, we assembly circRNAs using a circRNA-seq library of a pooled sample, which reduced the possible incorrectness caused by the noise of linear transcripts during circRNA assembly. CircRNAs could play roles by interacting with host genes [70]. Interestingly, our results showed that the host genes of Y2-selective circRNAs were involved in the lipid metabolic process, suggesting the potential regulatory mechanisms of the circRNAs regulating their host genes during BAT whitening. The functions and regulatory mechanisms of circRNAs transcribed from lipid-related genes warrant further interrogation. However, the investigation of the circRNA interacting with host genes is out of the scope of ceRNA regulation in this article. Taken together, these results represent the first to systematically identify the genome-wide circRNAs of BATs in rabbits. Considering the gain of the “WAT-like” phenotype of BAT, our circRNA set might represent common adipose circRNAs to some extent.

Canonically, RNA cyclization was considered a process that improves the stability of RNA to play its steady regulatory role [71]. While currently, the relationship between BSJs and miRNAs was rarely studied. In this study, we analyzed the possibility of the BSJ sequence of circRNA being the MRE of miRNA. Our data showed that the RNA cyclization improved the sponging capacity of the miRNAs that had been reported to be involved in regulating the thermogenesis of adipocytes, such as ocu-miR-378-5p, ocu-miR-433-3p, and ocu-miR-22-3p [49–51], indicating the RNA cyclization might be a critical event for sponging miRNAs during BAT whitening.

Many studies have revealed that circRNAs play roles by ceRNA mechanism, in which miRNAs play the center roles [72]. To further understand the impact of circRNA-related ceRNA crosstalk on BAT whitening, we combined miRNA-mRNA and miRNA-circRNA interaction data to construct the circRNA-miRNA-mRNA triple networks. Our data showed that circRNAs with different expression patterns formed various ceRNA networks. Although the D0-, D15-, and D85-selective circRNAs were predicted to regulate a small number of mRNAs via interacting miRNAs, they were found to be formed regulatory axes with key genes. For instance, the deficiency of *IDH2* was previously reported to lead to an obese-resistant phenotype and lower visceral fat accumulation, and inactivation of *IDH2* could activate molecules involved in stimulating energy expenditure in adipocytes [73]. Our prediction showed that a D15-selective circRNA (novel-circ-0005656) might activate the expression of *IDH2* by the ceRNA mechanism, suggesting the upregulation of novel-circ-0005656 might promote the BAT whitening by activating the *IDH2* during the early whitening stage. Y2-selective circRNAs were predicted to regulate the large number of mRNA via interacting with miRNAs. The adipose development-related signal pathway MAPK [60] singling pathway were significantly enriched by the Y2-selective circRNAs, which suggested that circRNAs might regulate final stage of BAT whitening via the MAPK signaling pathway by ceRNA mechanism.

Due to the great portion of miRNAs overlapping, the constructed lncRNA-miRNA-mRNA triple network was found to regulate similar biological processes with circRNA-miRNA-mRNA, indicating the cooperativity of circRNAs and lncRNAs in regulating BAT whitening by ceRNA mechanism. On the other hand, an analysis of the difference between circRNA-related ceRNA networks and lncRNA-related ceRNA networks found that 11 and 4 miRNAs specifically interacted with lncRNAs and circRNAs, respectively. Additionally, the 4 miRNAs that specifically interacted with circRNAs were all associated with adipogenesis, such as ocu-miR-140-3p [74], ocu-miR-184-3p [75], ocu-miR-25-3p [76], and ocu-miR-574-3p [77], which might suggest the importance of the circRNA-specific ceRNA networks in the development of BAT. In total, these data identified common and specific ceRNA networks between lncRNA and circRNA during the BAT whitening in rabbits. Based on the lncRNA expression patterns, we found that a small number of mRNAs at D0 and D15 and a large number of mRNAs at D85 and Y2 regulated by lncRNAs involved ceRNA networks, which might suggest that lncRNAs mainly regulate BAT whitening in the later stage of BAT whitening in rabbits. A previous study showed that *de novo* lipid (DNL) synthesis was a key biological process during BAT whitening in mice [12], and our previous study showed that DNL-related genes reached the highest expression levels at D85 during BAT whitening [29]. In this study, the most significantly enriched GO-BP terms by the D85-selective lncRNAs involved ceRNA networks was positive regulation of cell growth. Considering the elevated hormones and growth factors in puberty of rabbits at D85, the enrichment of positive regulation of cell growth suggested that lncRNAs might play a crucial role in promoting BAT whitening at puberty by regulating genes involved in cell growth via ceRNA mechanism. Ras signaling pathway is a key adipose development-related signal pathway [78]. Our data showed that the Y2-selective lncRNA-related ceRNA network was significantly enriched in the Ras signaling pathway. Additionally, previous study revealed that Ras [79] is an upstream activation protein of PI3K and MAPK and both of PI3K [60] and MAPK [61] were key proteins involved in adipogenesis. The enrichment of Ras signaling pathway indicated that lncRNAs might activate genes in Ras signaling pathway and indirectly activate PI3K and MAPK to regulate the final formation of the WAT-like phenotype of BAT by the ceRNA mechanism.

Finally, to confirm our interaction prediction, we validated a subnetwork that was predicted to be involved in immune response using dual-luciferase reporter assays. Our data suggested the robust results of the established ceRNA networks. The cooperativity of novel_circ_0013792 and MSTRG.16862.1 in regulating immune response-related gene *SLC15A3* by sponging ocu-miR-378-5p thus warrants further functional and mechanism validation.

## Conclusions

Taken together, this work represents the first to systematically identify the genome-wide miRNAs and circRNAs of BATs in rabbits. Integrated analysis of miRNA, circRNA, lncRNA, and mRNA data constructed circRNA- and lncRNA-related ceRNA networks. The circRNA and lncRNA showed high cooperativity in sponging miRNAs and were both involved in immune response-associated biological processes by ceRNA mechanism during BAT whitening. The Y2-selective circRNA-related ceRNA network and lncRNA-related ceRNA network might regulate the formation of the WAT-like phenotype of BAT via MAPK and Ras signaling pathways, respectively. Moreover, the results from dual-luciferase reporter assays verified the accuracy of our interaction prediction. In addition, the cyclization of circRNAs improving the capacity in sponging miRNA might be a novel potential regulatory mode in ceRNA regulation during BAT whitening. Therefore, the present study provides a framework for understanding the post-transcriptional regulation during the loss of thermogenic capacity and the gain of the “WAT-like” phenotype of BAT, which might provide new insight into the BAT-based obesity treatments.

## Supplementary Information

**Additional file 1:** **Figure S1.** Gene expression changes of adipose markers and principal components analysis (PCA) of RNA-seq data. (A) Heat map shows the gene expression changes of adipose markers. (B) PCA were conducted using RNA-seq TPM values. The PC1 and PC2 represent the first principal component and second principal component, respectively. The variance that each principal component can explain were showed in brackets.

**Additional file 2:** **Figure S2.** Length distribution of miRNA-seq library and expressed miRNAs. (A) The number of reads from one representative miRNA-seq library with different lengths. (B) The Venn diagram analysis of expressed miRNAs among different growth stage.

**Additional file 3:** **Figure S3.** The expression of miRNAs with most number of targets and target prediction of miRNAs in miRC2, miRC4, and miRC7. (A) The expression of miRNAs with most number of targets. (B - D) target prediction of miRNAs in miRC2, miRC4, and miRC7. The red and blue nodes show the miRNAs and mRNAs, respectively.

**Additional file 4:** **Figure S4.** Identification and characterization of circular RNAs (circRNAs) of BATs in rabbits. (A) Characteristics of circRNAs identified in a pooled RNA sample using circRNA-seq. (B) The number of two types of RNA splicing events in the whole-transcriptome data of BATs.

**Additional file 5:** **Figure S5.** The PCR products amplified by the divergent primers. The bands with expected length size were extracted and subjected to Sanger sequencing. The gels were cropped according to the red line.

**Additional file 6: Figure S6.** Differential analysis of circRNAs and host gene analysis. (A) The number of detected differentially expressed circRNAs (DECs) in different comparisons. The scatter plot showed circRNAs with | log2(fold-change) | > 1 in the six comparisons. The red and orange points show the circRNAs with a *p*-value < 0.05. (B) GO-BP enrichment of host genes of DECs. (C) KEGG pathway analysis of host genes of DECs.

**Additional file 7:** **Figure S7.** Prediction of ceRNA networks using stage-selective circRNAs and functional annotation. (A - D) Prediction of ceRNA networks using circRNAs in circRC5, circRC3, circRC6, and circRC7. The red, blue, and green nodes show the miRNAs, mRNAs, and circRNAs, respectively. (E) GO-BP enrichment and KEGG pathway analysis of circRC7 involved ceRNA network.

**Additional file 8:** **Figure S8.** Prediction of ceRNA networks using stage-selective lncRNAs. The red, blue, and purple nodes show the miRNAs, mRNAs, and lncRNAs, respectively. (A - D) LncRC7, lncRC3, lncRC4, and lncRC5 involved lncRNA-miRNA-mRNA networks, respectively.

**Additional file 9:** **Figure S9.** Validation of full length of novel_circ_0013792 using two pairs of the divergent primers. The bands with expected length size were extracted and subjected to Sanger sequencing. The gels were cropped according to the red lines.

**Additional file 10:** **Table S1.** Summary of miRNA-seq data and circRNA-seq data.

**Additional file 11:** **Table S2.** The expression of miRNAs.

**Additional file 12:** **Table S3.** Significantly enriched GO-BP terms by target genes of miRNAs in miRC2.

**Additional file 13:** **Table S4.** Significantly enriched KEGG pathways by target genes of miRNAs in miRC2.

**Additional file 14:** **Table S5.** Genomic annotation of identified circRNAs.

**Additional file 15:** **Table S6.** Primers used in RT-PCR.

**Additional file 16:** **Table S7.** CircRNA-miRNA-mRNA regulatory axes with circRNA conservation annotation.

**Additional file 17:** **Table S8.** LncRNA-miRNA-mRNA regulatory axes with lncRNA conservation annotation.

**Additional file 18:** **Table S9.** Significantly enriched immune response-associated GO-BP terms by circRNA-related ceRNA networks.

## Data Availability

The original data files have been uploaded and published to the NCBI SRA database. The accession number is PRJNA716375 (https://www.ncbi.nlm.nih.gov/bioproject/?term=PRJNA716375) and PRJNA854761 (https://www.ncbi.nlm.nih.gov/bioproject/PRJNA854761).
