# Supplementary material for: Integrated analysis of microRNAs, circular RNAs, long non-coding RNAs, and mRNAs revealed competing endogenous RNA networks involved in brown adipose tissue whitening in rabbits
Source: BMC Genomics. 2022 Nov 28;23:779. doi: 10.1186/s12864-022-09025-2 (PMC9703717; doi:10.1186/s12864-022-09025-2)
Supplement: Supplementary file 1 — Additional file 1: Figure S1. Gene expression changes of adipose markers and principal components analysis (PCA) of RNA-seq data. (A) Heat map shows the gene expression changes of adipose markers. (B) PCA were conducted using RNA-seq TPM values. The PC1 and PC2 represent the first principal component and second principal component, respectively. The variance that each principal component can explain were showed in brackets. [file 12864_2022_9025_MOESM1_ESM.pdf]

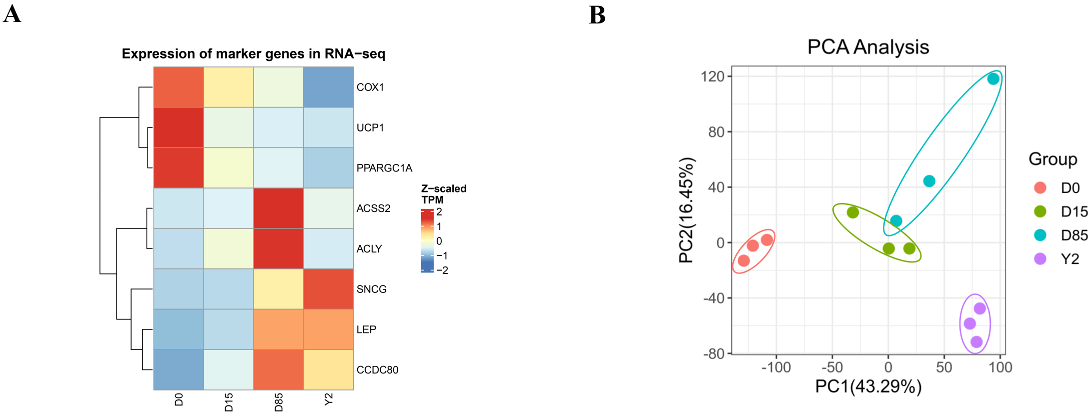

Figure S1. Gene expression changes of adipose markers and principal components analysis (PCA) of RNA-seq data. (A) Heat map shows the gene expression changes of adipose markers. (B) PCA were conducted using RNA-seq TPM values. The PC1 and PC2 represent the first principal component and second principal component, respectively. The variance that each principal component can explain were showed in brackets.
