# Supplementary material for: Integrated analysis of microRNAs, circular RNAs, long non-coding RNAs, and mRNAs revealed competing endogenous RNA networks involved in brown adipose tissue whitening in rabbits
Source: BMC Genomics. 2022 Nov 28;23:779. doi: 10.1186/s12864-022-09025-2 (PMC9703717; doi:10.1186/s12864-022-09025-2)
Supplement: Supplementary file 2 — Additional file 2: Figure S2. Length distribution of miRNA-seq library and expressed miRNAs. (A) The number of reads from one representative miRNA-seq library with different lengths. (B) The Venn diagram analysis of expressed miRNAs among different growth stage. [file 12864_2022_9025_MOESM2_ESM.pdf]

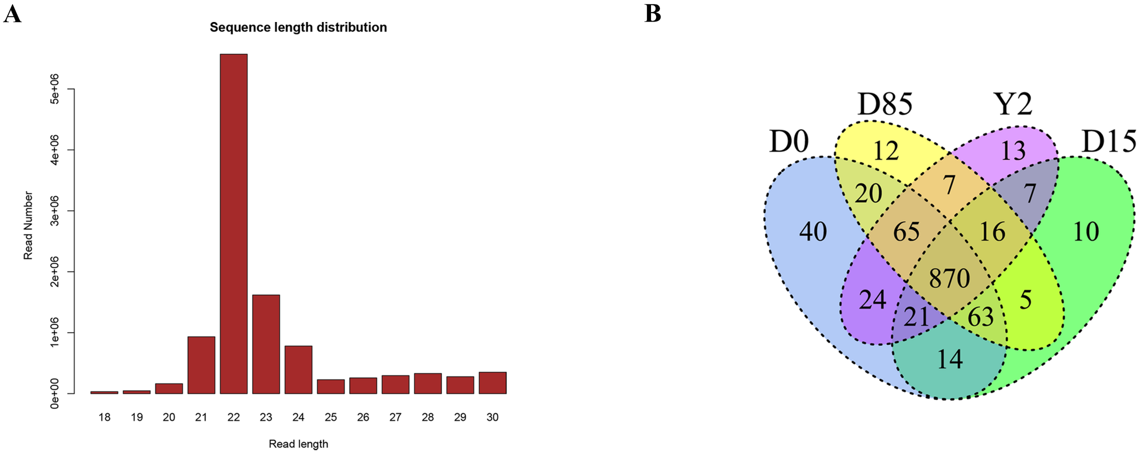

Figure S2. Length distribution of miRNA-seq library and expressed miRNAs. (A) The number of reads from one representative miRNA-seq library with different lengths. (B) The Venn diagram analysis of expressed miRNAs among different growth stages.
