# Supplementary material for: Integrated analysis of microRNAs, circular RNAs, long non-coding RNAs, and mRNAs revealed competing endogenous RNA networks involved in brown adipose tissue whitening in rabbits
Source: BMC Genomics. 2022 Nov 28;23:779. doi: 10.1186/s12864-022-09025-2 (PMC9703717; doi:10.1186/s12864-022-09025-2)
Supplement: Supplementary file 3 — Additional file 3: Figure S3. The expression of miRNAs with most number of targets and target prediction of miRNAs in miRC2, miRC4, and miRC7. (A) The expression of miRNAs with most number of targets. (B - D) target prediction of miRNAs in miRC2, miRC4, and miRC7. The red and blue nodes show the miRNAs and mRNAs, respectively. [file 12864_2022_9025_MOESM3_ESM.pdf]

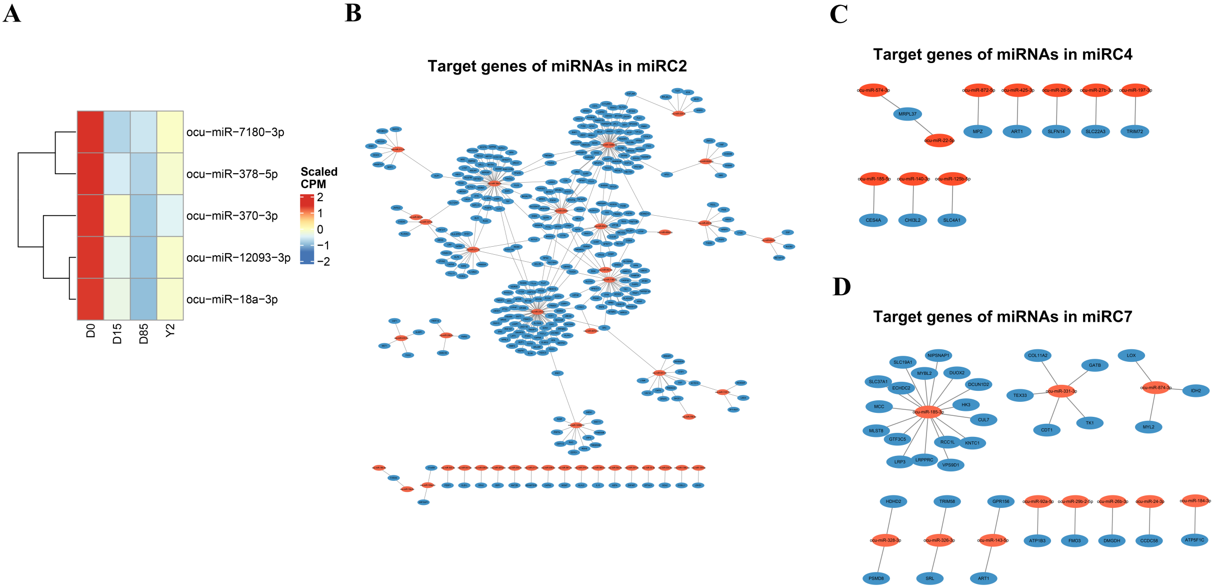

Figure S3. The expression of miRNAs with most number of targets and target prediction of miRNAs in miRC2, miRC4, and miRC7. (A) The expression of miRNAs with most number of targets. (B - D) target prediction of miRNAs in miRC2, miRC4, and miRC7. The red and blue nodes show the miRNAs and mRNAs, respectively.
