# Supplementary material for: Integrated analysis of microRNAs, circular RNAs, long non-coding RNAs, and mRNAs revealed competing endogenous RNA networks involved in brown adipose tissue whitening in rabbits
Source: BMC Genomics. 2022 Nov 28;23:779. doi: 10.1186/s12864-022-09025-2 (PMC9703717; doi:10.1186/s12864-022-09025-2)
Supplement: Supplementary file 4 — Additional file 4: Figure S4. Identification and characterization of circular RNAs (circRNAs) of BATs in rabbits. (A) Characteristics of circRNAs identified in a pooled RNA sample using circRNA-seq. (B) The number of two types of RNA splicing events in the whole-transcriptome data of BATs. [file 12864_2022_9025_MOESM4_ESM.pdf]

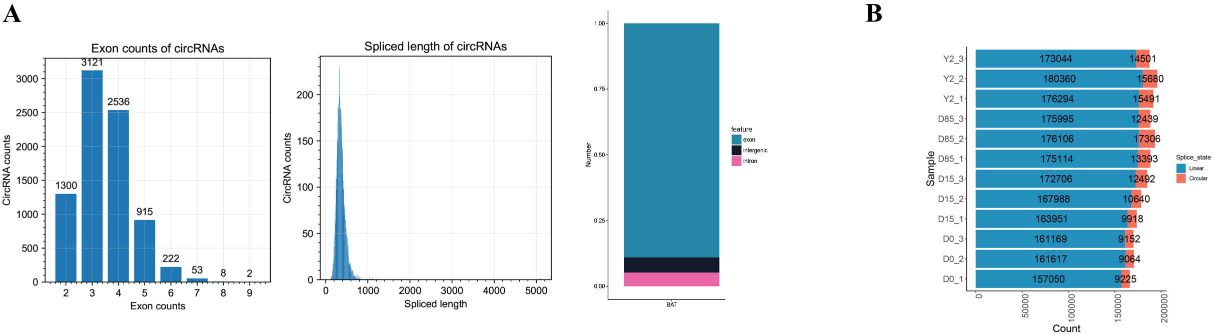

Figure S4. Identification and characterization of circular RNAs (circRNAs) of BATs in rabbits. (A) Characteristics of circRNAs identified in a pooled RNA sample using circRNA-seq. (B) The number of two types of RNA splicing events in the whole-transcriptome data of BATs.
