# Supplementary material for: Integrated analysis of microRNAs, circular RNAs, long non-coding RNAs, and mRNAs revealed competing endogenous RNA networks involved in brown adipose tissue whitening in rabbits
Source: BMC Genomics. 2022 Nov 28;23:779. doi: 10.1186/s12864-022-09025-2 (PMC9703717; doi:10.1186/s12864-022-09025-2)
Supplement: Supplementary file 5 — Additional file 5: Figure S5. The PCR products amplified by the divergent primers. The bands with expected length size were extracted and subjected to Sanger sequencing. The gels were cropped according to the red line. [file 12864_2022_9025_MOESM5_ESM.pdf]

Original gel images of figure 4D

The upper panel of figure 4D was cropped from red frame

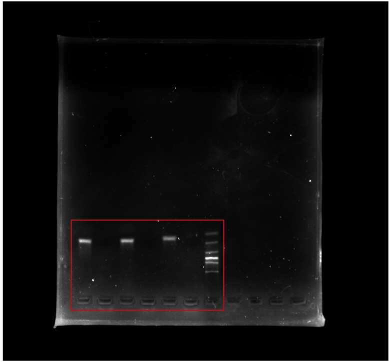

The middle panel of figure 4D was cropped from red frame

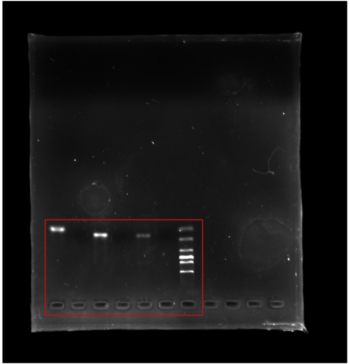

The bottom panel of figure 4D was cropped from red frame

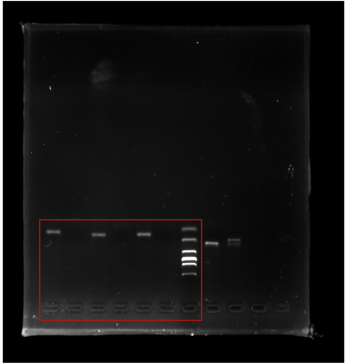

Figure S5. The PCR products amplified by the divergent primers. The bands with expected length size were extracted and subjected to Sanger sequencing. The gels were cropped according to the red lines.
