# Supplementary material for: Integrated analysis of microRNAs, circular RNAs, long non-coding RNAs, and mRNAs revealed competing endogenous RNA networks involved in brown adipose tissue whitening in rabbits
Source: BMC Genomics. 2022 Nov 28;23:779. doi: 10.1186/s12864-022-09025-2 (PMC9703717; doi:10.1186/s12864-022-09025-2)
Supplement: Supplementary file 6 — Additional file 6: Figure S6. Differential analysis of circRNAs and host gene analysis. (A) The number of detected differentially expressed circRNAs (DECs) in different comparisons. The scatter plot showed circRNAs with | log2(fold-change) | > 1 in the six comparisons. The red and orange points show the circRNAs with a p-value < 0.05. (B) GO-BP enrichment of host genes of DECs. (C) KEGG pathway analysis of host genes of DECs. [file 12864_2022_9025_MOESM6_ESM.pdf]

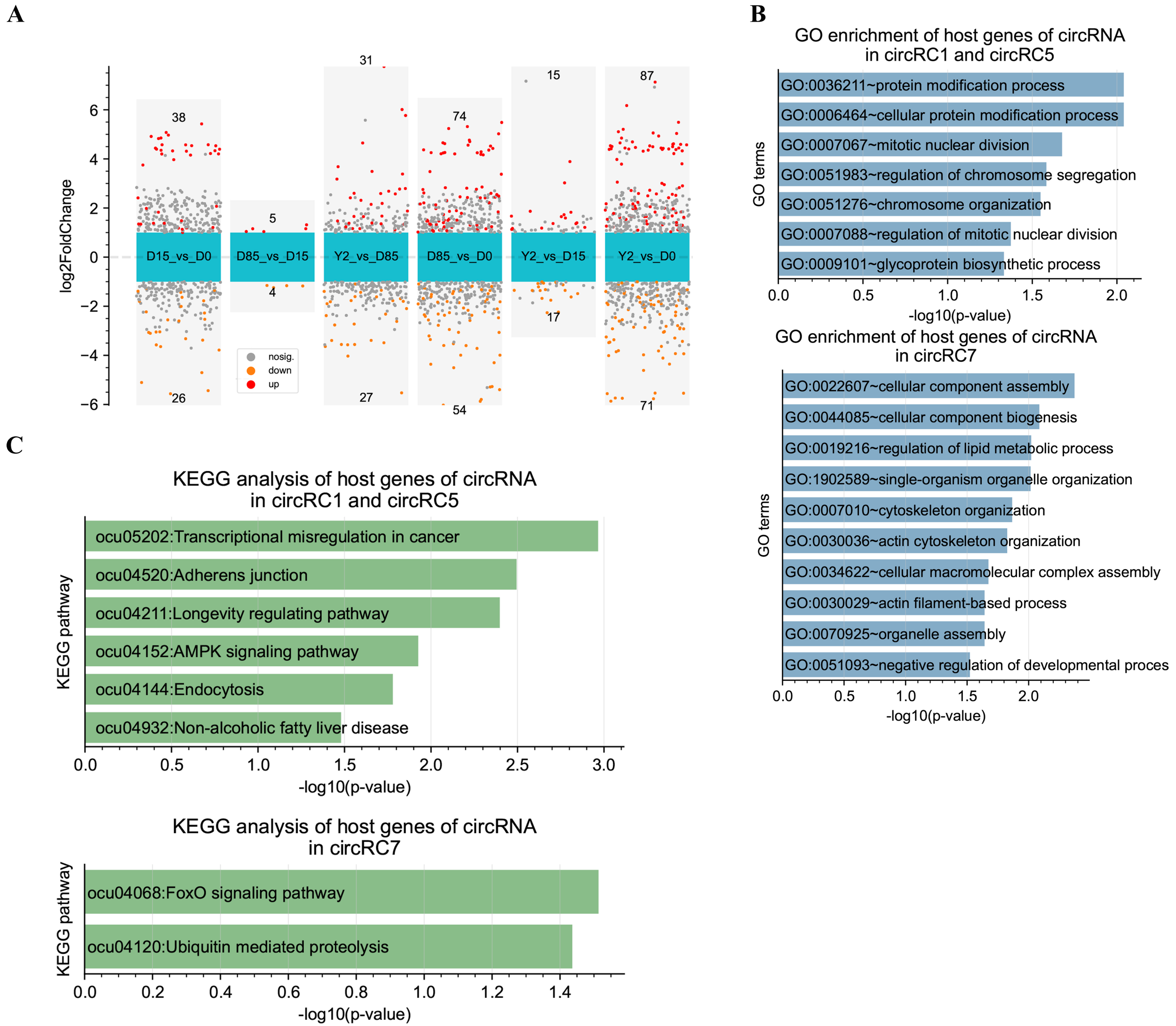

Figure S6. Differential analysis of circRNAs and host gene analysis. (A) The number of detected differentially expressed circRNAs (DECs) in different comparisons. The scatter plot showed circRNAs with $|\log2(\text{fold-change})| > 1$ in the six comparisons. The red and orange points show the circRNAs with a p-value < 0.05. (B) GO-BP enrichment of host genes of DECs. (C) KEGG pathway analysis of host genes of DECs.
