# Supplementary figures and images for: Integrated analysis of microRNAs, circular RNAs, long non-coding RNAs, and mRNAs revealed competing endogenous RNA networks involved in brown adipose tissue whitening in rabbits
Source: BMC Genomics. 2022 Nov 28;23:779. doi: 10.1186/s12864-022-09025-2 (PMC9703717; doi:10.1186/s12864-022-09025-2)

C

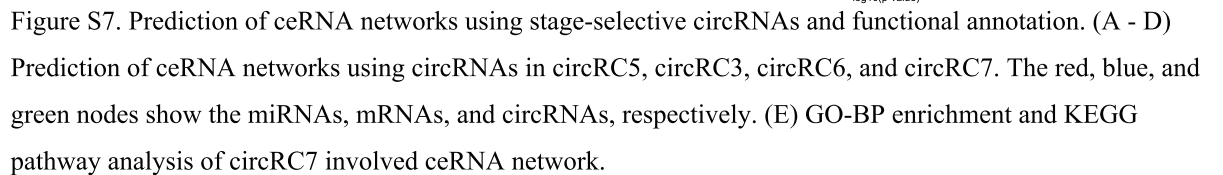

Supplement: Supplementary file 7 — Additional file 7: Figure S7. Prediction of ceRNA networks using stage-selective circRNAs and functional annotation. (A - D) Prediction of ceRNA networks using circRNAs in circRC5, circRC3, circRC6, and circRC7. The red, blue, and green nodes show the miRNAs, mRNAs, and circRNAs, respectively. (E) GO-BP enrichment and KEGG pathway analysis of circRC7 involved ceRNA network. [file 12864_2022_9025_MOESM7_ESM.pdf]
