# Supplementary material for: Integrated analysis of microRNAs, circular RNAs, long non-coding RNAs, and mRNAs revealed competing endogenous RNA networks involved in brown adipose tissue whitening in rabbits
Source: BMC Genomics. 2022 Nov 28;23:779. doi: 10.1186/s12864-022-09025-2 (PMC9703717; doi:10.1186/s12864-022-09025-2)
Supplement: Supplementary file 8 — Additional file 8: Figure S8. Prediction of ceRNA networks using stage-selective lncRNAs. The red, blue, and purple nodes show the miRNAs, mRNAs, and lncRNAs, respectively. (A - D) LncRC7, lncRC3, lncRC4, and lncRC5 involved lncRNA-miRNA-mRNA networks, respectively. [file 12864_2022_9025_MOESM8_ESM.pdf]

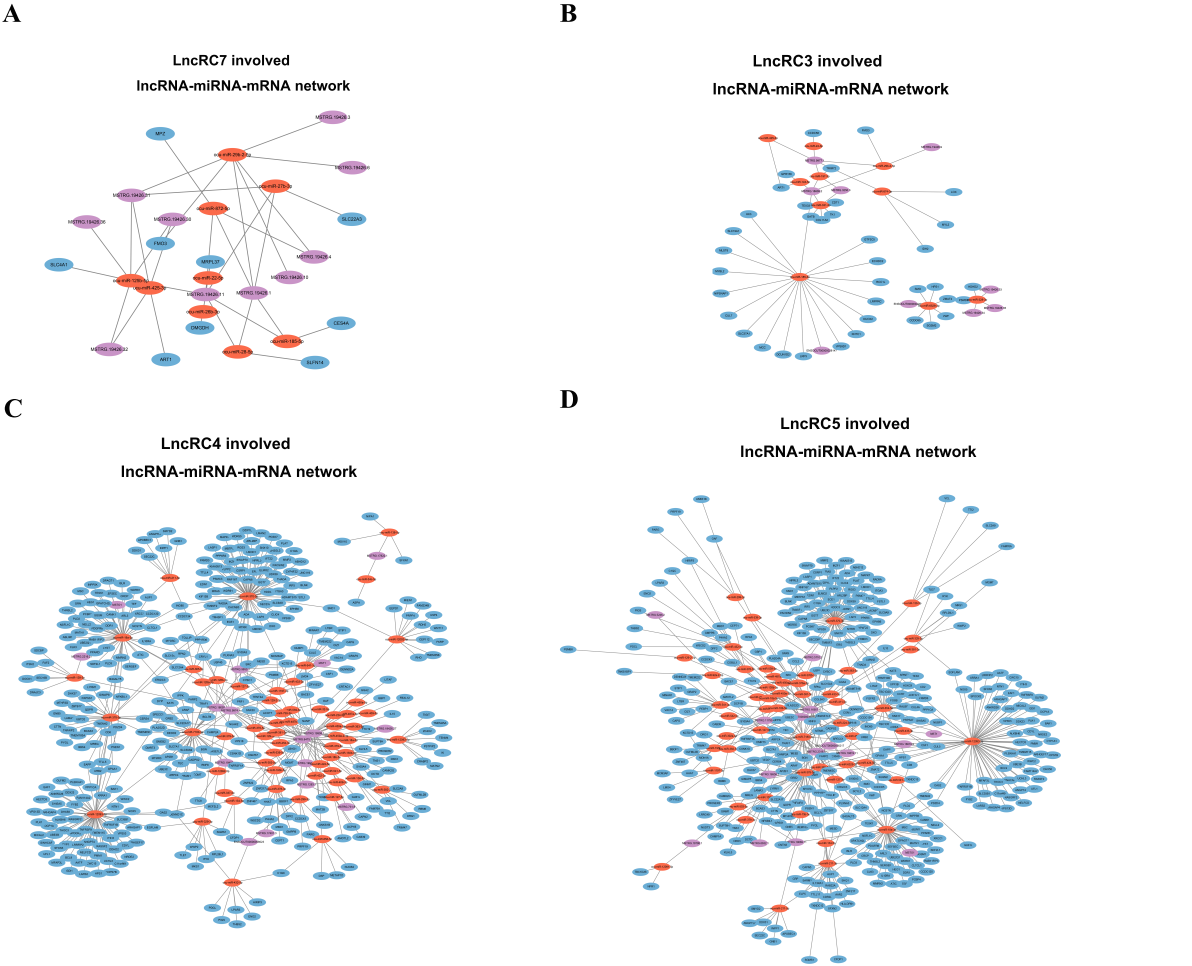

Figure S8. Prediction of ceRNA networks using stage-selective lncRNAs. The red, blue, and purple nodes show the miRNAs, mRNAs, and lncRNAs, respectively. (A - D) LncRC7, lncRC3, lncRC4, and lncRC5 involved lncRNA-miRNA-mRNA networks, respectiviely.
