# Supplementary material for: Integrated analysis of microRNAs, circular RNAs, long non-coding RNAs, and mRNAs revealed competing endogenous RNA networks involved in brown adipose tissue whitening in rabbits
Source: BMC Genomics. 2022 Nov 28;23:779. doi: 10.1186/s12864-022-09025-2 (PMC9703717; doi:10.1186/s12864-022-09025-2)
Supplement: Supplementary file 9 — Additional file 9: Figure S9. Validation of full length of novel_circ_0013792 using two pairs of the divergent primers. The bands with expected length size were extracted and subjected to Sanger sequencing. The gels were cropped according to the red lines. [file 12864_2022_9025_MOESM9_ESM.pdf]

## Original gel imags for figure 7D

The left panel of figure 7D was cropped from red frame

The right panel of figure 7D was cropped from red frame

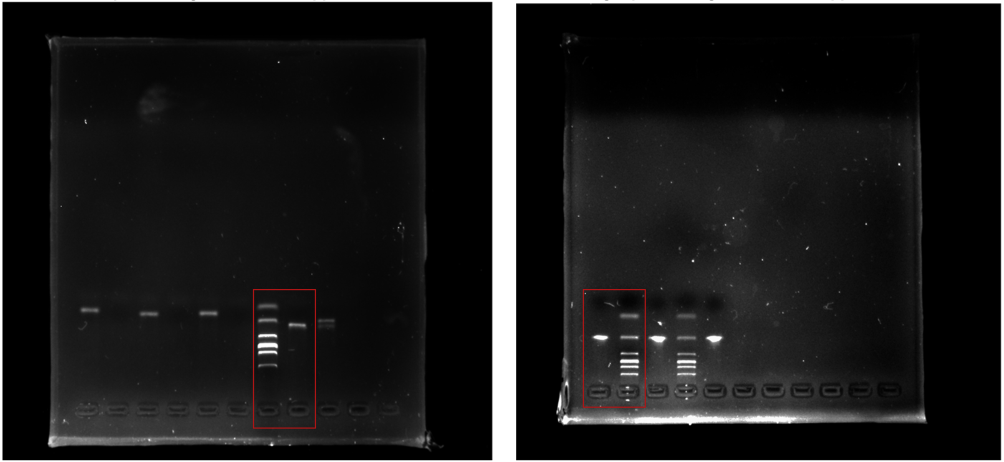

Figure S9. Validation of full length of novel_circ_0013792 using two pairs of the divergent primers. The bands with expected length size were extracted and subjected to Sanger sequencing. The gels were cropped according to the red lines.
