# Supplementary material for: Integrated analysis of microRNAs, circular RNAs, long non-coding RNAs, and mRNAs revealed competing endogenous RNA networks involved in brown adipose tissue whitening in rabbits
Source: BMC Genomics. 2022 Nov 28;23:779. doi: 10.1186/s12864-022-09025-2 (PMC9703717; doi:10.1186/s12864-022-09025-2)
Supplement: Supplementary file 10 — Additional file 10: Table S1. Summary of miRNA-seq data and circRNA-seq data. [file 12864_2022_9025_MOESM10_ESM.docx]

**Table S1 Summary of miRNA-seq data and circRNA-seq data**

| **Library type** | **Sample** | **Group** | **reads** | **bases** | **Q20** | **Q30** | **GC content** |
| --- | --- | --- | --- | --- | --- | --- | --- |
| MiRNA-seq | D0_1 | D0 | 10904637 | 2.52E+08 | 98.50% | 95.70% | 45.00% |
| MiRNA-seq | D0_2 | D0 | 17792090 | 3.9E+08 | 99.40% | 97.70% | 45.00% |
| MiRNA-seq | D0_3 | D0 | 20745308 | 4.54E+08 | 99.40% | 98.00% | 45.30% |
| MiRNA-seq | D15_1 | D15 | 10263982 | 2.59E+08 | 99.20% | 97.30% | 46.80% |
| MiRNA-seq | D15_2 | D15 | 14461862 | 3.39E+08 | 99.30% | 97.20% | 44.20% |
| MiRNA-seq | D15_3 | D15 | 15976973 | 3.73E+08 | 99.30% | 97.30% | 42.40% |
| MiRNA-seq | D85_1 | D85 | 20234500 | 4.44E+08 | 99.50% | 97.90% | 43.80% |
| MiRNA-seq | D85_2 | D85 | 12000199 | 2.7E+08 | 99.30% | 97.50% | 43.20% |
| MiRNA-seq | D85_3 | D85 | 9814595 | 2.36E+08 | 99.30% | 97.30% | 45.20% |
| MiRNA-seq | Y2_1 | Y2 | 16566489 | 3.65E+08 | 99.30% | 97.30% | 43.80% |
| MiRNA-seq | Y2_2 | Y2 | 16043487 | 3.53E+08 | 99.40% | 97.70% | 45.30% |
| MiRNA-seq | Y2_3 | Y2 | 9623771 | 2.26E+08 | 99.30% | 97.30% | 46.00% |
| CircRNA-seq | Pooled sample | - | 105100316 | 1.58E+10 | 96.65% | 91.85% | 65.49% |
