# Supplementary material for: Integrated analysis of microRNAs, circular RNAs, long non-coding RNAs, and mRNAs revealed competing endogenous RNA networks involved in brown adipose tissue whitening in rabbits
Source: BMC Genomics. 2022 Nov 28;23:779. doi: 10.1186/s12864-022-09025-2 (PMC9703717; doi:10.1186/s12864-022-09025-2)
Supplement: Supplementary file 15 — Additional file 15: Table S6. Primers used in RT-PCR. [file 12864_2022_9025_MOESM15_ESM.docx]

**Table S6 Primers used in RT-PCR**

| **Number** | **Used in** | **Type** | **Gene ID** | **Gene name** | **Primer (forward/reverse)** | **Product length (bp)** |
| --- | --- | --- | --- | --- | --- | --- |
| 1 | Validation of cyclization | Divergent | *Novel_circ_0017105* | *GL018827_575864_577014(+)* | GGTCCCTCCTTGATCCGGT/TGCTCTAGGAATGGACGACT | 158 |
| 2 | Validation of cyclization | Divergent | *Novel_circ_0009771* | *2_28764150_28764661(+)* | GAGAACGGCTGGTTCACAGAC/CTTTCTGATGCGGGCGGAAAT | 146 |
| 3 | Validation of cyclization | Divergent | *Novel_circ_0011014* | *3_66631696_66632130(-)* | GGGAAGTGGAACCGGATATTTA/GCTTCCAATAATGTAGTGTGGTTTG | 116 |
| 4 | Validation of cyclization | Divergent | *Novel_circ_0006569* | *18_49713965_49723592(-)* | ACCATCCAGACTGAAGACCG/GAGACACCTTCACCCGTTCC | 94 |
| 5 | Validation of cyclization | Divergent | *Novel_circ_0007366* | *19_49695291_49696242(-)* | CACTGGCATTAGCCATTTTGGA/TGTTGATATTTCCTTTCATGACCA | 161 |
| 6 | Validation of cyclization | Divergent | *Novel_circ_0007513* | *1_106364219_106364974(-)* | GCCTGAATCTCCGGTGCAA/AGCCCATGAAGCTGTTCCG | 169 |
| 7 | Validation of cyclization | Divergent | *Novel_circ_0011546* | *4_48194015_48207625(+)* | GATCGGGACCGAGAACGTG/CTGGGGCATCGCCATTATTT | 133 |
| 8 | Validation of cyclization | Divergent | *Novel_circ_0011771* | *4_79390717_79395740(-)* | TGCTACCCAAAGTTCACATCTTG/ATTGGGGATGGGCCATTAGGA | 162 |
| 9 | Validation of cyclization | Divergent | *Novel_circ_0013792* | *8_78806219_78807533(-)* | GTGAAGAGAGTTTCAGCATCAGT/GCAGACGGTGGAAGGTATGT | 147 |
| 9 | Amplifying Full length | Divergent | *Novel_circ_0013792* | *8_78806219_78807533(-)* | TCAATTCCTCCACCAGAGCAAC/GCTTGAACTTGAGACTCCAGCA | 305 |
| 9 | Amplifying Full length | Divergent | *Novel_circ_0013792* | *8_78806219_78807533(-)* | AAATGGCCCTAAGATGCTGGAG/GGAAGGTATGTGGTAGGGGAT | 251 |
